# Silica–Lignin Composites—Insight into Physicochemical Properties and Adsorption Mechanisms

**DOI:** 10.3390/ijms27219954

**Published:** 2026-11-06

**Authors:** Magdalena Blachnio, Malgorzata Zienkiewicz-Strzalka, Zofia Juchniewicz, Dariusz Sternik, Anna Derylo-Marczewska

**Affiliations:** Department of Physical Chemistry, Institute of Chemical Sciences, Maria Curie-Sklodowska University, Maria Curie-Sklodowska Square 3, 20-031 Lublin, Poland; malgorzata.zienkiewicz-strzalka@mail.umcs.pl (M.Z.-S.); zofia_juchniewicz@interia.pl (Z.J.); dariusz.sternik@umcs.pl (D.S.)

**Keywords:** silica–lignin composites, mesoporous materials, organic dyes, adsorption kinetics, adsorption equilibrium

## Abstract

Two silica–lignin composites with varying biopolymer content were synthesized using the sol–gel method as effective adsorbents for the removal of organic dyes from aqueous solutions. The textural, structural, and surface properties of the synthesized materials were determined using nitrogen adsorption–desorption techniques, XRD, XPS, and FTIR. XPS and XRD analysis confirmed the effective modification of the silica surface with lignin. Increasing the lignin content in the composite altered its physicochemical properties. Textural analysis revealed a decrease in the porosity of the composites with increasing the amount of biopolymer incorporated into the silica. Thermal analysis with the identification of gaseous thermal destruction products allowed for the determination of the thermal stability of the composites. Dye adsorption experiments, including equilibrium and kinetic measurements, allowed the determination of adsorption capacity, adsorption rate and adsorption mechanisms. FTIR mapping enabled the identification of differences in the interactions of selected dyes with the synthesized composites and revealed preferential adsorption on surface areas of the adsorbents rich in lignin or silica. The obtained results confirmed the potential of silica–lignin materials in environmental applications as cheap and biodegradable adsorbents.

## 1. Introduction

Composite and hybrid materials that combine the advantages and reduce the disadvantages of pure materials are gaining increasing interest in many areas of industry, medicine, and ecology [1,2,3,4,5,6,7]. Such composites consist of at least two phases, and as a result of the synergistic effect, a material with properties different from those of the individual components is obtained. Materials composed of at least one biocomponent are particularly attractive due to their lower cost, availability of renewable natural resources, biocompatibility, and biodegradability [8]. Ecologically sustainable technologies based on new eco-friendly materials can reduce environmental pollution and improve living conditions for humans and animals. Biopolymers widely used in the production of new types of composites include, among others, starch, lignin and cellulose [9,10,11,12,13,14].

Lignin is a renewable biopolymer that is attracting increasing attention across multiple industrial sectors. It is a natural material that binds cellulose units, which can be separated and chemically modified. Derived from lignocellulosic biomass and generated as a major byproduct of pulping and biorefinery processes, lignin has become an important research target for applications in composite systems, functional (active) materials, and packaging, as well as for use as an additive in polymeric and bio-based matrices. This interest is driven in part by lignin’s capacity to modulate the physicochemical properties of host materials, including their mechanical, thermal, barrier, and surface characteristics. In addition, lignin offers several practical and functional advantages, including wide availability, comparatively low feedstock cost, a favorable environmental profile, intrinsic antioxidant and antimicrobial activity, biodegradability, and reinforcing potential [15,16,17]. Lignin is an abundant, heterogeneous biopolymer characterized by a complex, cross-linked three-dimensional architecture. It is covalently associated with hemicellulose primarily through ester linkages. Lignin biosynthesis proceeds via the radical polymerization of three principal monolignols: p-coumaryl, coniferyl, and sinapyl alcohols which contribute to key material attributes, including mechanical recalcitrance and thermal stability. Nevertheless, lignin reactivity and macroscopic behavior are governed predominantly by the nature and abundance of reactive functional moieties within the polymer, notably aliphatic and phenolic hydroxyl, carboxyl, and phenolic groups. The distribution and concentration of these functionalities vary as a function of botanical origin and the processing or isolation methodology [18].

Lignin-based composites are formulated by blending lignin in variable proportions with other lignocellulosic fibers and bio-derived additives (e.g., waxes). In some systems, lignin is additionally combined with petroleum-derived constituents; however, the primary objective of lignin composite development is to provide environmentally preferable alternatives to conventional plastics. Owing to its aromatic, multifunctional macromolecular architecture, lignin can serve as a filler, stabilizing agent, compatibilizer, and reinforcing phase in composite matrices. Lignin is also frequently subjected to chemical modification to tailor material physicochemical properties, including enhanced mechanical performance and thermal stability. Notably, even in its unmodified form, lignin may confer functionality such as ultraviolet (UV) stabilization, antioxidant activity, and plasticization, depending on formulation and processing conditions. A representative case is poly(lactic acid) (PLA)/lignin biocomposite, which have been reported to exhibit high antioxidant activity, favorable melt-processability (including extrudability and melt-flow behavior), and broad applicability in packaging; biomedical and hygiene products; agricultural materials; water-treatment media; and additive manufacturing (3D printing) [19,20,21].

In medical applications, particularly tissue engineering approaches involving transplantation, repair, and structural support for tissue or organ regeneration, electrospun nanofibrous membranes based on lignin and polycaprolactone (PCL), as well as 3D-printed lignin-containing hydrogels, and various lignin-based systems have been investigated. These uses exploit lignin’s antioxidant, antimicrobial, immunomodulatory, and biocompatibility-related properties [22,23,24,25,26]. In a separate study, the performance of biocomposites comprising modified lignin and TOFA (tall oil fatty acid) incorporated into poly(vinyl alcohol) cross-linked with citric acid was evaluated. The resulting material formed a stable, compatible matrix and exhibited significant improvements in tensile strength and thermal resistance, supporting potential use in packaging, automotive components, construction and insulation materials, and electronic devices [27]. Specially designed, multifunctional lignin-based materials may find new applications as: lignin-phenol-formaldehyde resin-based adhesives, flocculants, lignin-plastic composites, and carbon materials—including fibers and carbon dots—derived from lignin [28]. Review [29] discusses lignin-based materials, which exhibit diverse properties depending on the biopolymer’s source, the extraction process, and chemical modifications aimed at enhancing its reactivity. Potential applications for these biocomposites—characterized by improved properties and reduced environmental impact—include the food and packaging sectors, seed coating, the automotive industry, 3D printing, biomedical applications, the rubber industry, and the production of wood adhesives.

A cross-linked material was prepared from carboxylated lignin, poly(vinyl alcohol) (PVA), and vanillin using Ca^2+^ ions as the cross-linking agent. The resulting material exhibited pronounced hydrophobicity and biodegradability, enhanced mechanical performance, and high resistance to UVB and UVC irradiation, while effectively attenuating most UVA radiation. This material was subsequently applied as a coating for paper substrates [30]. In addition, silica–lignin composites, characterized by favorable dispersion and morphology, good electrokinetic (colloidal) stability, suitable thermal properties, and a porous microstructure, represent a promising alternative for use as polymer fillers or as adsorbents for hazardous chemical species, including heavy metals [31]. Actual publications offer an excellent example of an ecological, zero-waste approach in preparation lignin based composites. Unbleached corn cob and wheat straw, often wasted and incinerated as agricultural waste, was used to produce lignin-containing cellulose biocomposites (LCB). These materials are distinguished by their exceptional mechanical strength, UV-protective properties, and environmental friendliness, as well as the radiation-protective properties of cellulose/lignin composites in which azide-modified cellulose dissolved in dimethylacetamide/lithium chloride (DMAc/LiCl) was reacted with propargylated lignin, resulting in biodegradable and flexible UV-protective films [32,33].

Given the properties of lignin and the potential applications of materials based on this biopolymer—as outlined above—extensive research is being conducted on the synthesis and characterization of various composites involving different solid phases. However, to design optimal materials and understand the processes occurring within the pore system and on the composite surfaces, it is crucial to conduct research on a wide range of such systems. In this study, silica–lignin composites were synthesized and characterized, and their suitability as adsorbents for organic pollutants in water treatment technologies was investigated. Two key factors determining the efficiency of adsorption techniques—adsorption capacity and adsorption rate—were analyzed in detail based on equilibrium and kinetic measurements. To assess the influence of the composites’ textural and surface properties on adsorption processes, various experimental techniques were employed; these enabled a comprehensive characterization of the materials and an analysis of the interactions between the adsorbates and the surfaces of the different solid phases.

## 2. Results and Discussion

### 2.1. Textural, Structural, and Surface Properties of Silica–Lignin Composites

**Figure 1:**
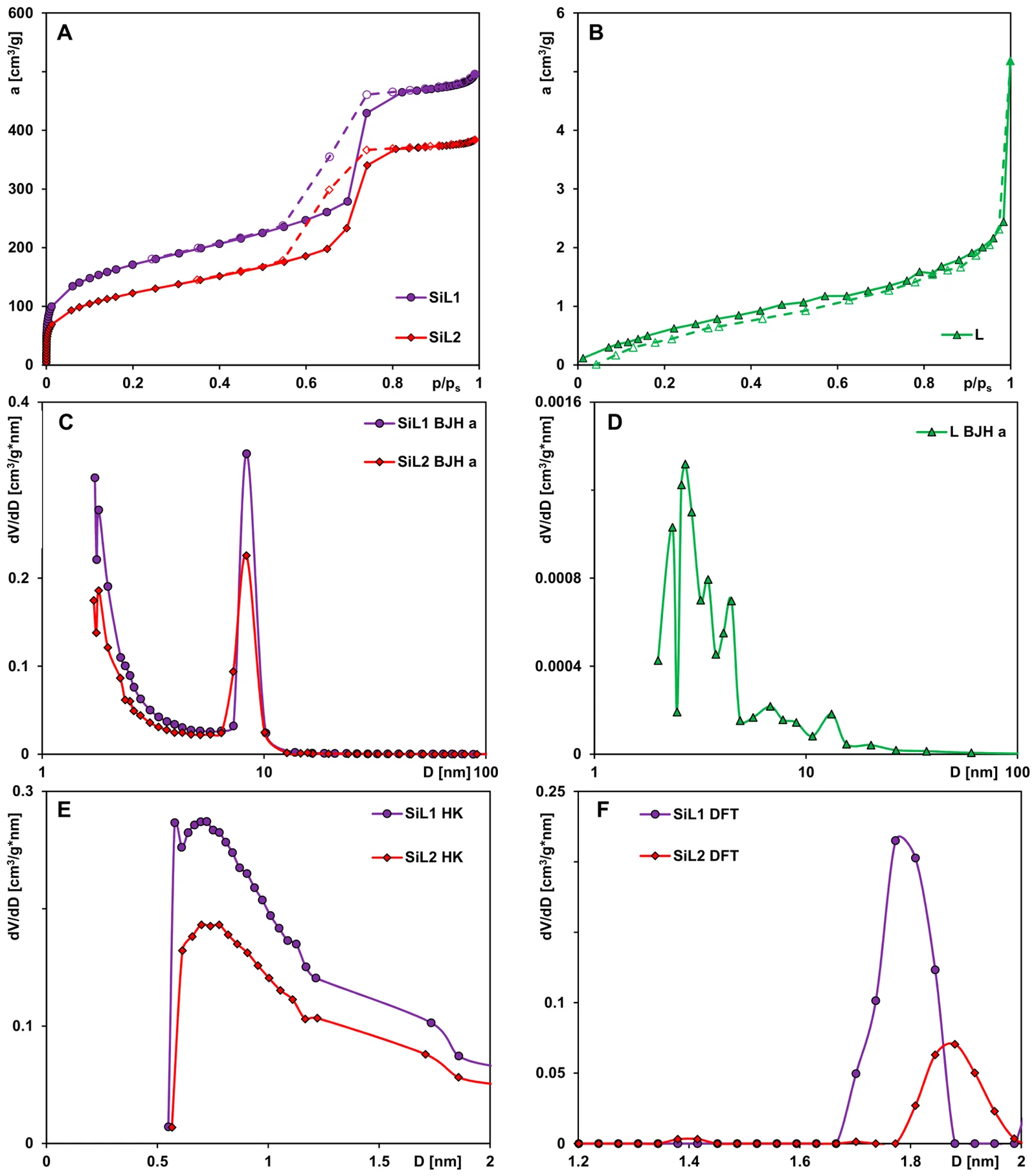
(**A**) Nitrogen adsorption–desorption isotherms for the silica–lignin composites SiL1, SiL2, (**B**) lignin, (**C**,**D**) BJH pore size distributions determined from the adsorption branch of isotherms, (**E**,**F**) HK and DFT pore size distributions determined from the adsorption branch of isotherms.

Figure 1A–F present the nitrogen adsorption–desorption isotherms and the corresponding pore size distribution functions determined for the silica–lignin composites and native lignin. The adsorption isotherms of the composites are classified as type IV according to the IUPAC classification, indicating the mesoporous nature of the synthesized materials (Figure 1A). The relatively low nitrogen uptake at low relative pressures suggests the formation of only a limited number of micropores during the synthesis process, whereas the pronounced increase in adsorption at relative pressures above *p*/*p*_0_ > 0.5 indicates the development of a well-defined mesoporous structure. The high nitrogen uptake observed at a relative pressure of *p*/*p*_0_~0.98 reflects the formation of an internal porous structure with a substantial pore volume. The pore size distribution curves determined by the BJH method for both composites confirm the high uniformity of the obtained mesoporous structure (the narrow peaks within the small mesopore range, with maxima located at approximately 8 nm and 5–6 nm based on the adsorption branches of the isotherms) (Figure 1C). The pore size distributions in the range of micropores determined by HK and DFT methods confirm the formation of a small population of micropores with maxima below 2 nm (Figure 1E,F). The nitrogen adsorption–desorption isotherm of native lignin corresponds to type II according to the IUPAC classification, which is characteristic of nonporous materials (Figure 1B). The narrow hysteresis loop indicates the presence of only a small fraction of mesopores, while the negligible nitrogen uptake at low relative pressures confirms the absence of micropores. The pore size distribution curves exhibit numerous peaks, indicating that lignin contains a small population of pores with a broad size distribution. The well-developed porous structure of both composites and the essentially nonporous character of the biopolymer are further confirmed by the textural parameters, i.e., the specific surface area and total pore volume (Table 1). The results demonstrate that the textural properties of the composites strongly depend on the lignin content. Increasing the amount of non-porous biopolymer in the reaction mixture (a 3-fold higher lignin content used during the synthesis of the SiL2 composite compared with SiL1) resulted in a decrease both in the specific surface area from 616 to 444 m^2^ g^−1^ and in the total pore volume from 0.76 to 0.60 cm^3^ g^−1^. At the same time, the change in the components ratio was accompanied by a marked increase in the contribution of mesopores to the overall pore structure, from 0.76 for SiL1 to 0.92 for SiL2. The relatively low external surface area of both composites (24–48 m^2^ g^−1^) indicates a high degree of pore structure uniformity, which is consistent with the pore size distribution curves.

**Figure 2:**
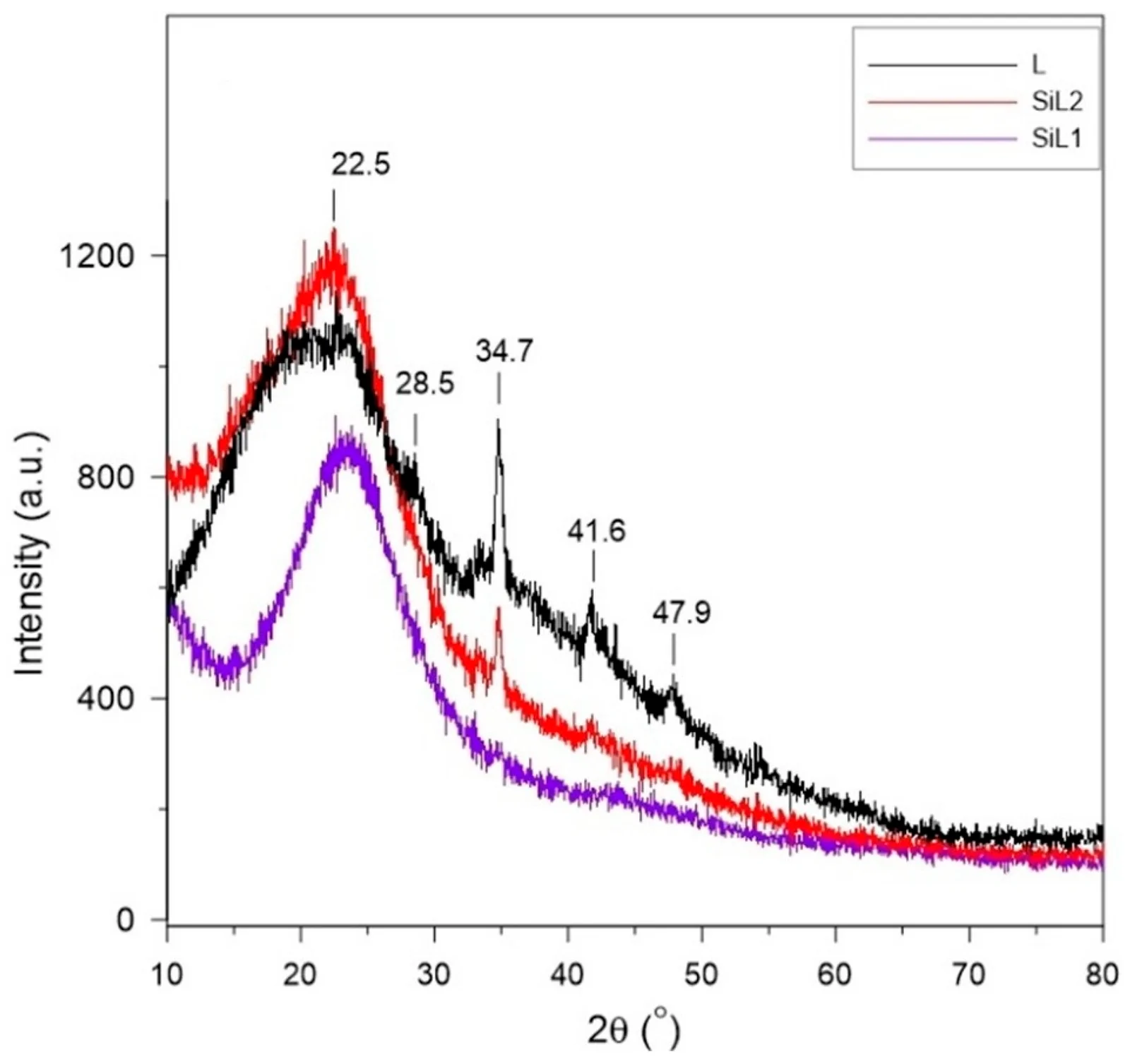
X-ray diffraction (XRD) diffractograms of lignin (L) and silica–lignin composites (SiL1 and SiL2).

Lignin, as a heterogeneous biopolymer formed from phenylpropane units connected by random ether and C–C bonds, is characterized by a random, three-dimensional, and highly branched crosslinked network. As a result of radical coupling of the three main monolignols, a system is formed that does not exhibit repeating structural sequences. Such spatial irregularity, in turn, leads to the formation of an irregular crystalline lattice. The diffraction curves shown in Figure 2 illustrate the phase structure of the lignin substrate (L) and the lignin-silica composites SiL1 and SiL2.

The XRD pattern of alkali lignin exhibits a broad diffraction halo centered at approximately 22.5° together with several weak reflections at higher diffraction angles, indicating the presence of short-range ordered domains within the lignin material. Across all diffractograms of SiL1 and SiL2 composite materials, a broad intensity maximum centered at approximately 2θ ≈ 22.5° is also observed. This feature is characteristic of predominantly amorphous lignin-based materials and reflects the presence of short-range structural ordering within the organic matrix. The pronounced broadening of this diffraction maximum indicates the absence of extensive long-range crystalline organization. For the composite samples (SiL1 and SiL2), a diffraction maximum is likewise present within the 2θ ≈ 22–23° region. In these materials, the broad feature results from overlapping contributions of the lignin matrix and the amorphous silica phase, whose disordered SiO_4_ tetrahedral network gives rise to diffuse scattering in the same angular range. Consequently, all investigated materials exhibit a broad diffraction halo centered near 22.5°, consistent with the predominance of amorphous structural domains.

In addition to the principal amorphous contribution, the diffraction pattern of the lignin substrate (L) exhibits several weak reflections located at approximately 2θ = 28.5°, 34.7°, 41.6°, and 47.9°. The presence of these reflections suggests that the material contains a limited fraction of locally ordered domains inherited from the biomass precursor [34,35,36].

For the lignin-silica composites, the detectability of these reflections decreases relative to the lignin substrate. This effect may be attributed to the increasing contribution of the amorphous silica phase, which enhances diffuse scattering and reduces the apparent contrast of weak diffraction features originating from ordered domains. As a result, the diffraction patterns of SiL1 and SiL2 are dominated by the broad amorphous halo, confirming that the incorporation of silica increases the overall structural disorder of the hybrid materials. The absence of additional sharp Bragg reflections further indicates that no significant crystalline silica phases were formed during composite preparation.

**Figure 3:**
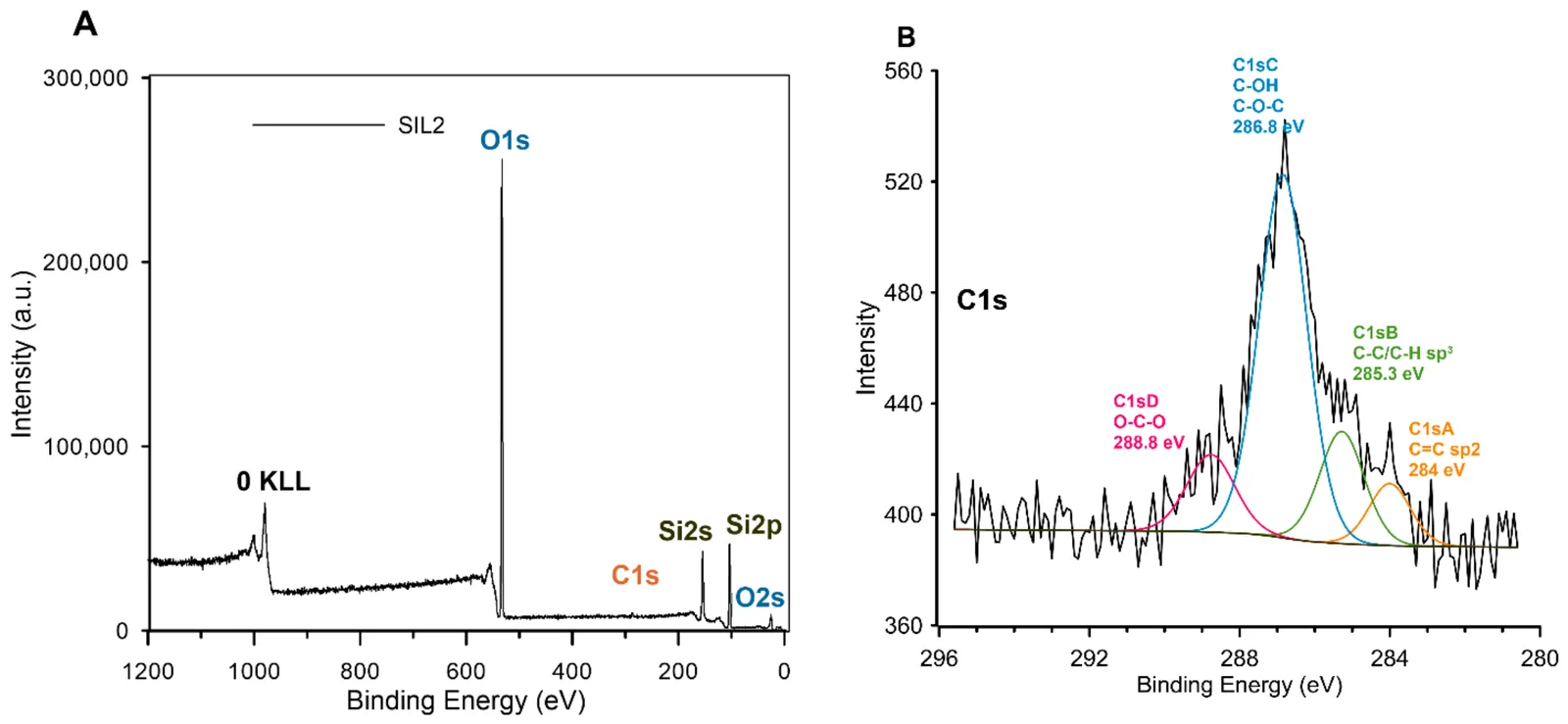
(**A**) Full XPS spectrum of the lignin-modified silica sample, (**B**) high-resolution XPS spectrum of carbon atoms.

Analysis of the full XPS survey spectrum of the lignin-modified silica sample provides direct evidence of successful functionalization and allows evaluation of chemical modifications within the near-surface region. The survey spectrum (Figure 3A) contains only signals attributable to Si, O, and C, indicating that no extraneous elements were introduced during synthesis or post-synthetic modification and supporting the high chemical purity of the resulting composite (Table 2). The dominant O 1s feature at 532.5 eV is consistent with the oxygen-rich character of silica. It is also compatible with oxygen-containing lignin functionalities (e.g., phenolic, aliphatic hydroxyl, and carbonyl groups), which are relevant to adsorption performance by increasing surface chemical heterogeneity and the density of potential interaction sites for dye molecules.

**Table 2:** XPS results for silica–lignin composite (SiL2). Positions of general peaks as a signal of photoelectrons from various energetic levels, and properties of XPS peaks from high resolution analysis and their assignment.

Name		Peaks Position (eV)	Full Width at Half Maximum (FWHM)	Atomic Concentration [%]	Mass Concentration [%]
Si 2p		103.5	2.40	37.40	52.00
O 1s		532.5	2.30	56.10	43.20
C 1s		286.0	2.57	6.50	4.80
High Resolution Analysis					assigned as
Si 2p		103.8	1.7	100	SiO_2_
O 1s		532.9	1.63	100	SiO_2_. C-O
C 1s	C 1s A	284	1.3	10.0	C=C
	C 1s B	285.3	1.4	17.5	C-C, C-H
	C 1s C	286.8	1.5	60.6	C-O
	C 1s D	288.8	1.5	11.9	O-C-O-

The Si 2p (103.5 eV) and week Si 2s (~155 eV) peaks confirm silicon predominantly in the oxidized Si^4+^ environment characteristic of the SiO_2_ lattice. Notably, the Si2s/O1s atomic content ratio increases from the value expected for stoichiometric silica (~0.5) to 0.68 after modification, indicating an apparent relative depletion of oxygen in the outermost analyzed layer. This shift is consistent with partial coverage of the silica surface by lignin, which attenuates contributions from the underlying Si–O network and thereby alters the detected elemental ratio. The C 1s signal at 286 eV further substantiates incorporation of organic matter at the surface; its intensity and envelope are consistent with lignin-derived aromatic/aliphatic carbon and oxidized carbon species. The measured carbon concentration (6.5 at.%) supports partial, rather than complete, coverage of the silica surface by a lignin overlayer, indicating effective functionalization while preserving exposure of the silica matrix. Additional features, including the O KLL Auger signal and a weak O 2s peak, are consistent with oxygen present in both the silica framework and lignin moieties. The absence of detectable signals from other elements corroborates the lack of undesired impurities, which is essential for subsequent adsorption studies and for mechanistic interpretation of dye—surface interactions. Overall, the atomic percentages (O: 56.1%, Si: 37.4%, C: 6.5%) confirm that lignin modifies the chemistry of the near-surface region without forming a continuous film, a configuration expected to be beneficial for adsorption by combining the porous silica substrate with lignin functional groups to enable synergistic binding interactions.

High-resolution X-ray photoelectron spectroscopy (XPS) provides chemically specific information on the surface atomic environments of lignin-functionalized silica, thereby enabling assessment of structural and electronic modifications induced by the functionalization procedure. Evaluation of the O 1s and Si 2p core-level regions indicates retention of the silica framework together with the emergence of interfacial interactions attributable to lignin incorporation.

The O 1s spectrum (Figure S1) is characterized by a single, well-resolved signal with a maximum at 532.9 eV. This value is close to the binding energy commonly reported for oxygen in stoichiometric SiO_2_ (approx. 533.2 eV). A slight shift toward lower binding energies (approx. 0.3 eV) may suggest a change in the local electronic environment of the surface oxygen species. Such a decrease in energy may be consistent with the partial involvement of oxygen atoms in C–O bonds and associated with functional groups derived from lignin which is expected when silica–lignin bonds are formed. From an electronic perspective, the XPS results illustrate the presence of lignin-derived organic functionalities within the surface region of the silica–lignin composite, indicating successful surface modification. However, the available data do not allow for an unambiguous distinction between covalent bonding and strong interfacial adsorption. This can lead to a reduction in the effective positive charge on the oxygen atoms, resulting in a lower measured O 1s binding energy; this trend is consistent with the differences in the electronegativity of the elements (χ_C_ = 2.55; χ_O_ = 3.44).

The Si 2p spectrum (Figure S1) exhibits a single signal at 103.8 eV, which is in agreement with the data in the literature for fully oxidised silicon (the Si^4+^ state) within a SiO_2_ structure [37]. The absence of additional signals in the Si 2p spectrum indicates that the silicon remains primarily in the Si^4+^ oxidation state. There is no spectroscopic evidence for the reduction of silicon or the formation of chemically distinct silicon species (such as Si–C bonds or significantly modified Si–OH groups). Furthermore, the presence of a single Si 2p signal suggests that the structure of the siloxane network remains intact. The preservation of the SiO_2_ framework is advantageous for the properties of the compound, as it maintains the inherent chemical stability and porosity of the silica, which is characteristics that are crucial for the subsequent use of the material as an adsorbent, for example, in the removal of dyes.

**Figure 4:**
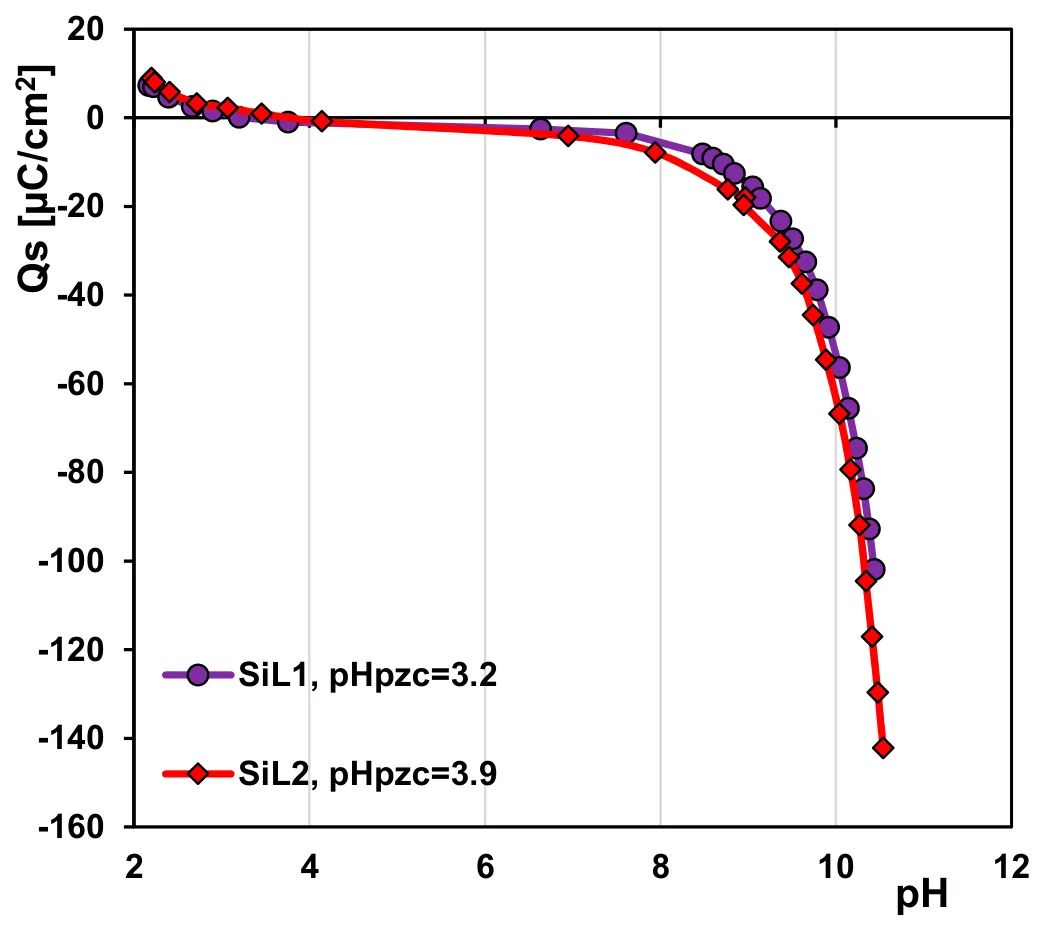
Dependences of surface charge density vs. pH for the SiL1 and SiL2 composites.

The acid-base surface properties of the lignin–silica composites were characterized by potentiometric titration. The relationship between the surface charge density and solution pH is presented in Figure 4. Based on the titration data, the point of zero charge (PZC) was determined to be 3.2 for SiL1 and 3.9 for SiL2. The PZC values are of particular importance in adsorption studies, as they govern the electrostatic interactions between the adsorbent surface and ionic pollutants present in the solution. When oppositely charged species are present, attractive electrostatic forces promote the adsorption process and facilitate the accumulation of adsorbate molecules on the composite surface. In contrast, the presence of the same sign charges may lead to electrostatic repulsion, reducing the affinity of the adsorbate toward the adsorbent.

**Figure 5:**
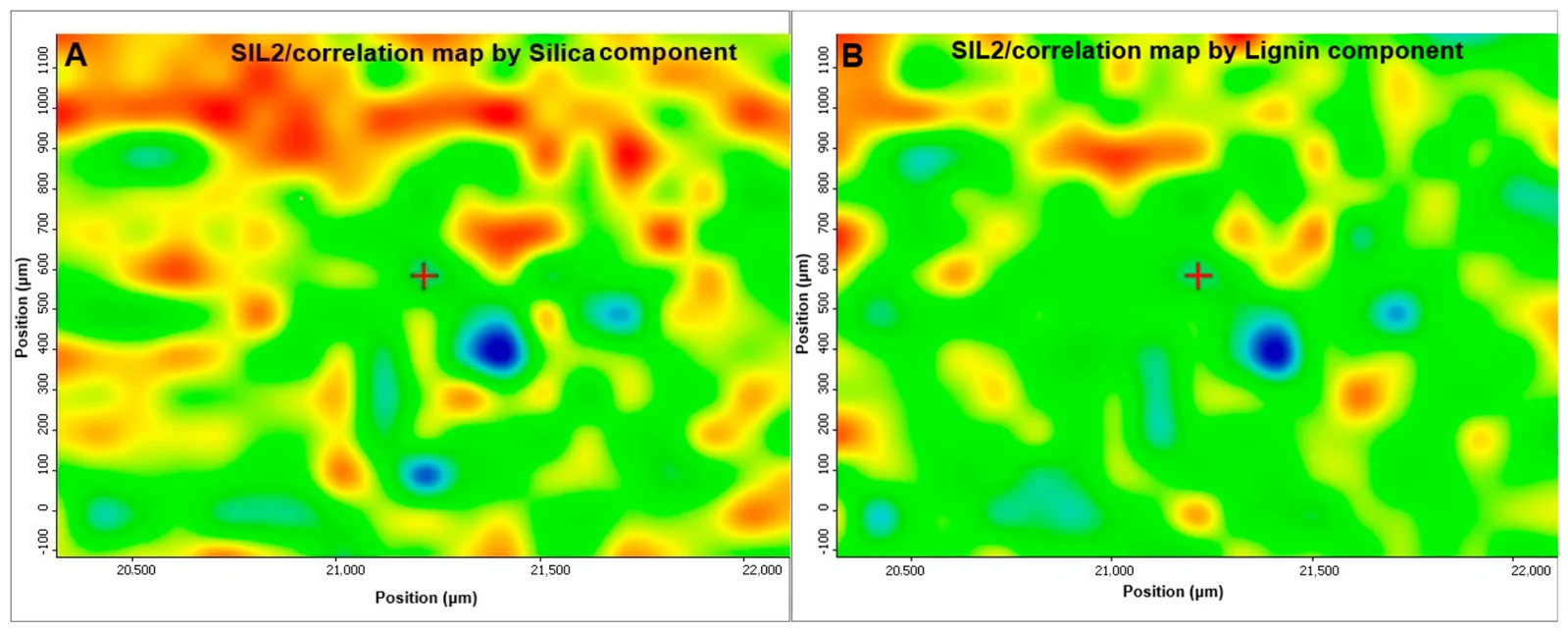
Fourier-transform infrared (FTIR) correlation maps of the silica–lignin composite, referenced to spectral bands characteristic of silica (**A**) and lignin (**B**). The color scale denotes the local correlation intensity, thereby delineating spatial variations in the distribution of each component and quantifying the extent of their co-localization within the composite microstructure.

FTIR mapping (Fourier transform infrared mapping) is an analytical approach for assessing the spatial distribution of chemical functionalities across the surface of lignin–silica composites through acquisition and interpretation of localized infrared spectra. During FTIR mapping, spectra are collected sequentially at discrete points, thereby generating a spatially resolved chemical dataset. Characteristic absorption bands are attributable to specific functional groups and following appropriate preprocessing and multivariate or peak-intensity analysis, band intensities or component scores can be converted to color scales to yield correlation maps that delineate the surface localization of particular composite constituents. Figure 5 shows correlation maps covering the SiL2 composite material systems with respect to the silica component and with respect to lignin. Similar correlation maps were also examined for the systems after dye adsorption and correlated with the components, including the dyes used. These results are presented in the section of the work concerning adsorption aspects.

Spatial correlation mapping of FTIR data for the silica–lignin composite demonstrates pronounced heterogeneity in the spatial distribution and co-localization of spectral features attributable to each constituent. Correlation maps referenced to silica and lignin exhibit distinct intensity patterns, consistent with a microstructurally diversified composite and spatially non-uniform interfacial interactions. In the silica-referenced correlation map, extended regions of high correlation are observed predominantly in the central area of the analyzed surface. The dominance of red and yellow regions indicates elevated and internally consistent silica-associated FTIR responses within these domains. This pattern is indicative of comparatively homogeneous silica dispersion and suggests the formation of quasi-continuous silica-rich domains within the composite. Conversely, zones of reduced correlation at the map periphery likely reflect local variations in silica packing density, plausibly arising from particle agglomeration and/or heterogeneous wetting of the lignin surface during composite formation.

By contrast, the lignin correlation map exhibits pronounced spatial heterogeneity. Highly correlated areas are sparse and predominantly point-like, whereas most of the analyzed surface shows low to moderate correlation values. This pattern is consistent with lignin representing only a minor fraction of the composite mass where its spectral signal is correspondingly weaker and appears confined to localized domains. The observed localization implies that lignin does not constitute a continuous phase; instead, it is present as discrete microaggregates and/or irregular deposits embedded within the silica matrix. Comparative analysis of the lignin and silica correlation maps further indicates incomplete spatial co-localization, i.e., lignin does not fully follow the spatial distribution of silica. Although lignin-containing domains occur within the silica framework, their arrangement does not replicate that of the inorganic phase, suggesting that lignin is immobilized or anchored to silica in a non-uniform manner. Overall, these observations support a composite microstructure in which lignin immobilization on silica yields local lignin-enriched regions, while the bulk of the material remains dominated by the silica phase. The heterogeneity of the lignin distribution likely reflects both its low loading and intrinsic physicochemical features of the biopolymer, including a propensity for aggregation and/or preferential deposition in specific microenvironments of the matrix. Conversely, the comparatively uniform correlation pattern for silica is consistent with effective silica formation and the structural stability of the inorganic network.

**Figure 6:**
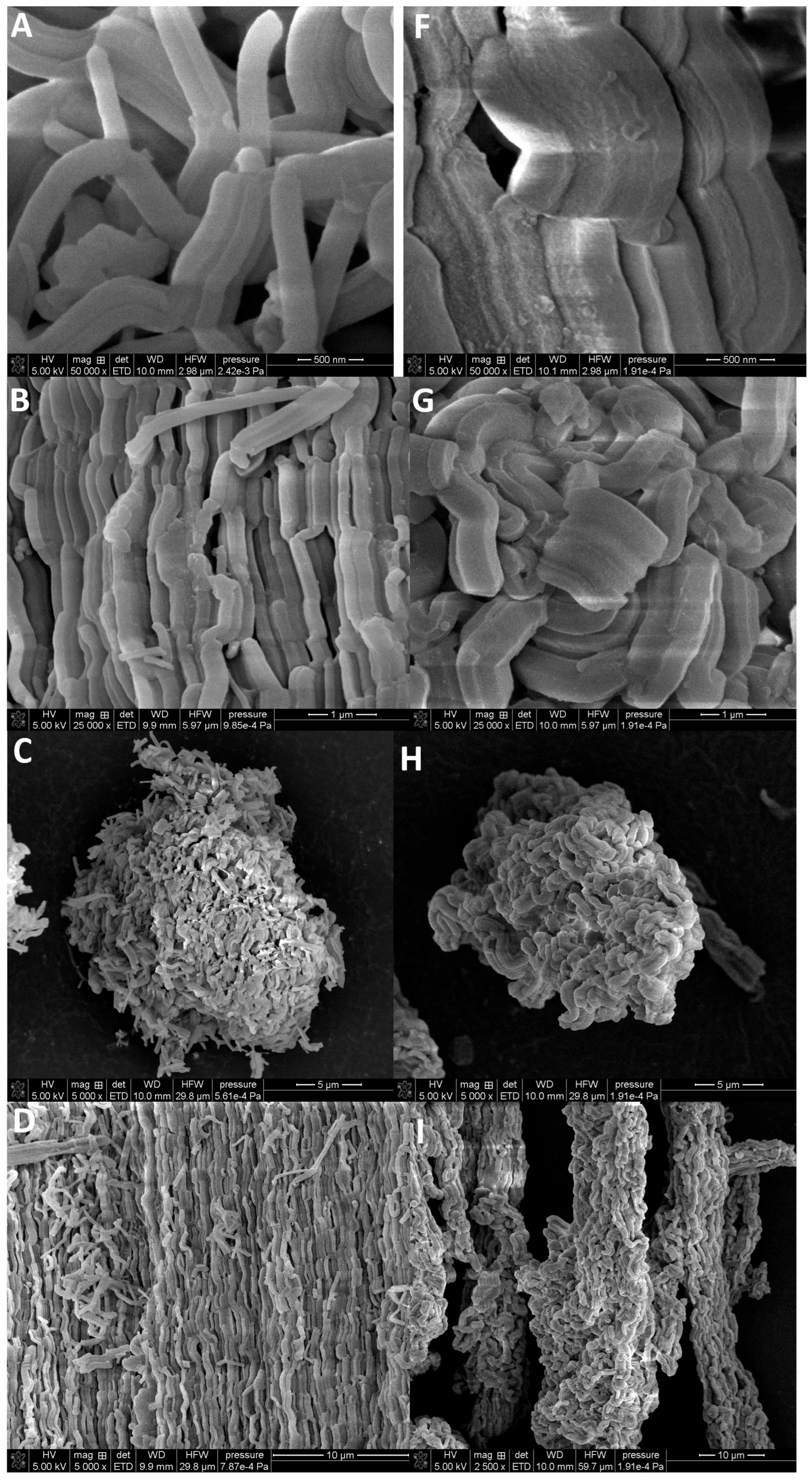

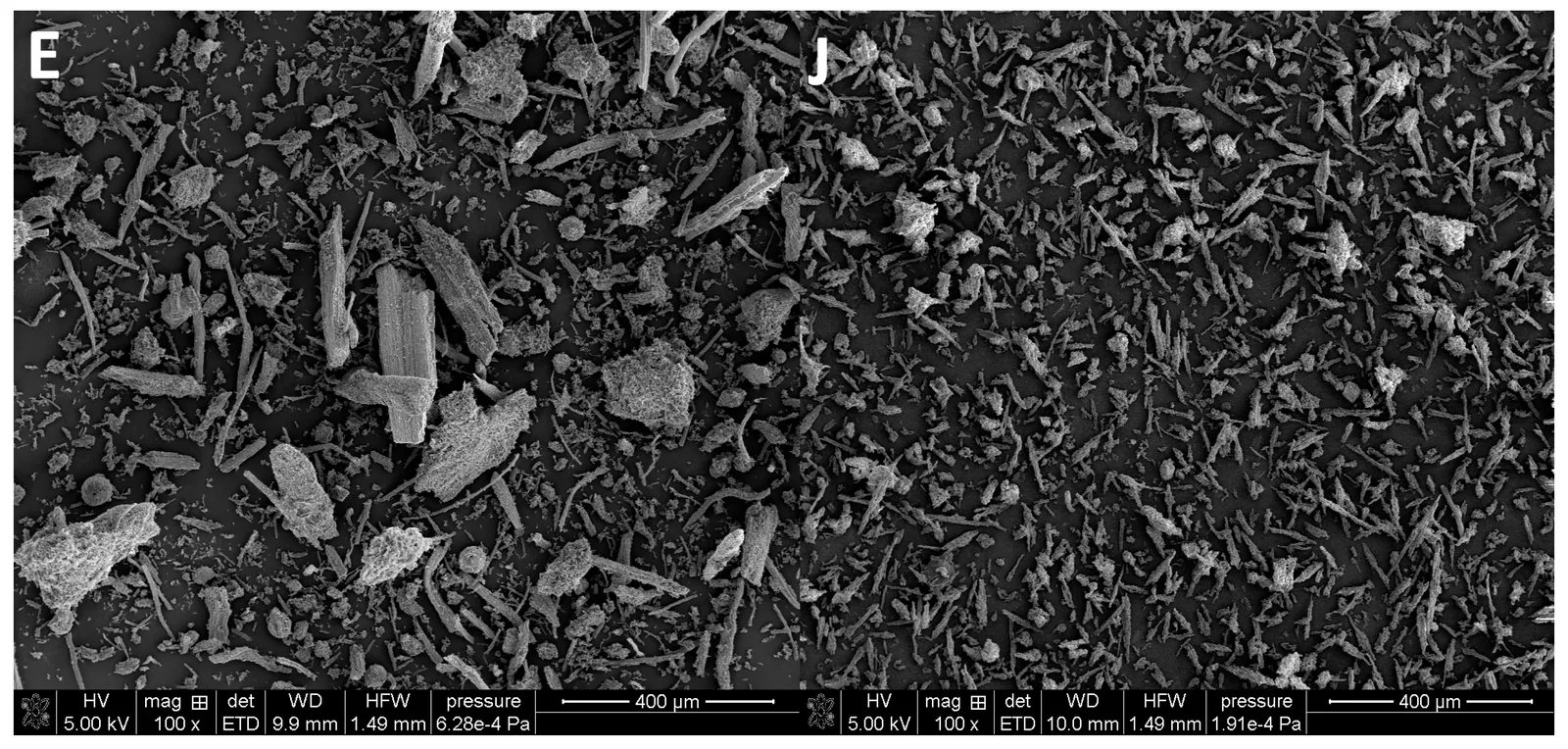
SEM images of silica–lignin composites SiL1 (**A**–**E**) and SiL2 (**F**–**J**), showing the effect of lignin content on the material morphology.

Scanning electron microscopy (SEM) micrographs (Figure 6A–E) characterize the morphology of the silica–lignin composite SiL1 across multiple length scales, evidencing a complex hierarchical architecture. At the nanometer scale (Figure 6A), irregular corrugated fibrous domains and amorphous plate-like features are observed, forming a dense, three-dimensional network. At intermediate magnifications (Figure 6B–D), the material exhibits more ordered, parallel arrangements with a lamellar morphology characteristic of silica-based structures. Numerous interlayer connections and bridging features are apparent, suggesting pronounced interfacial interactions and effective integration between the inorganic and organic components. At lower magnification (Figure 6E), the sample is composed of fragmented composite particles with a broad distribution of sizes and shapes, consistent with the intrinsic heterogeneity of the material. Upon increasing the lignin content (SiL2), the morphology transitions from relatively regular plate-like domains to more irregular, corrugated, and amorphous structures (Figure 6F–J). The lamellae become thicker and less uniform, and surface roughness increases, consistent with enhanced coverage/coating of the inorganic phase by the organic fraction. In addition, spherical to irregular agglomerates attributable to lignin coalescence are detected, together with fibrous features associated with increased porosity. Collectively, these observations indicate a shift from a predominantly silica-governed morphology toward a more organic-rich, mechanically compliant, and heterogeneous composite structure.

### 2.2. Thermal Properties of Silica–Lignin Composites

**Figure 7:**
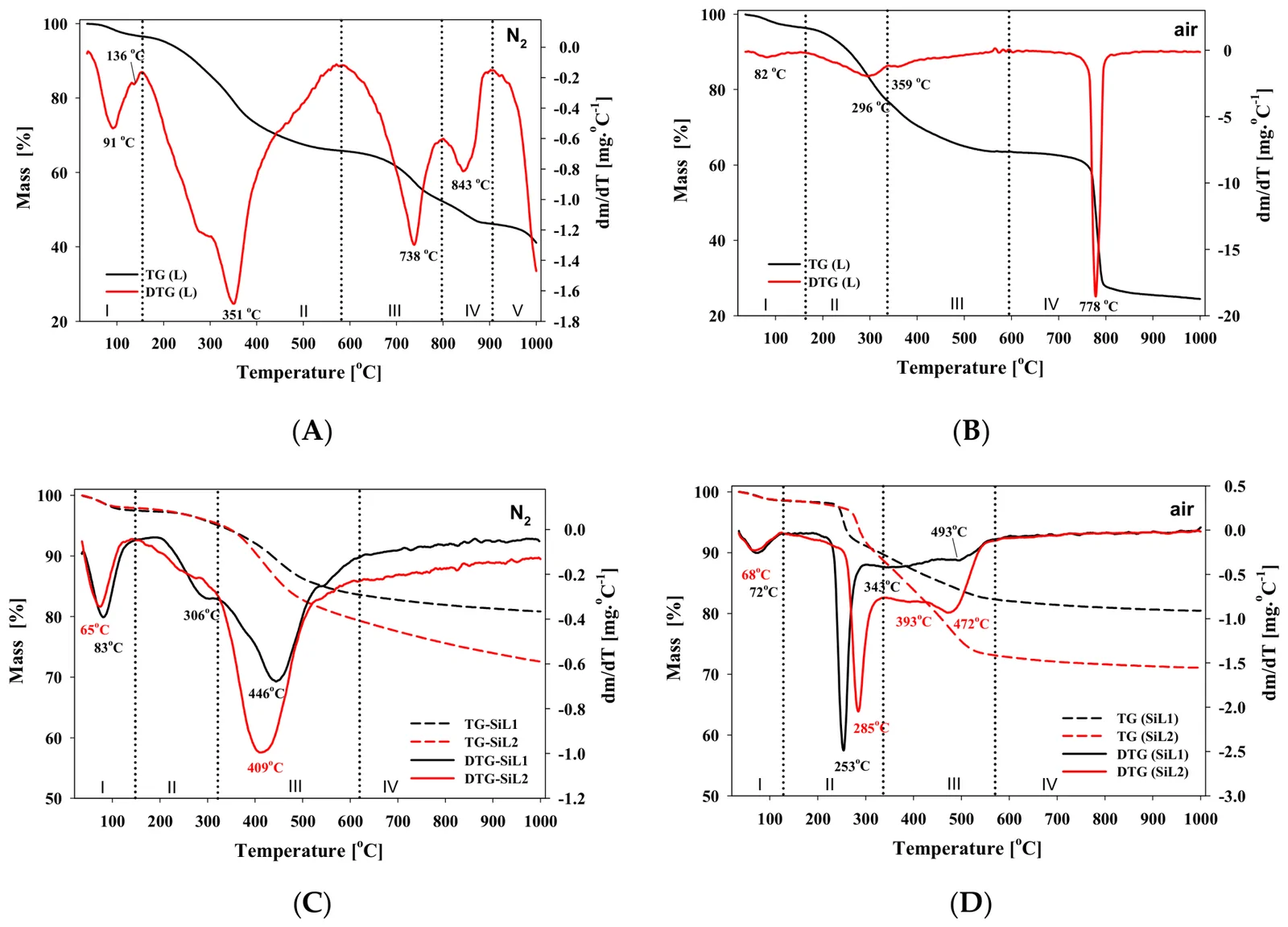
TG–DTG (**A**,**B**) curves of lignin and TG–DTG (**C**,**D**) curves of composites heated in nitrogen (**A**,**C**) and air (**B**,**D**).

**Figure 8:**
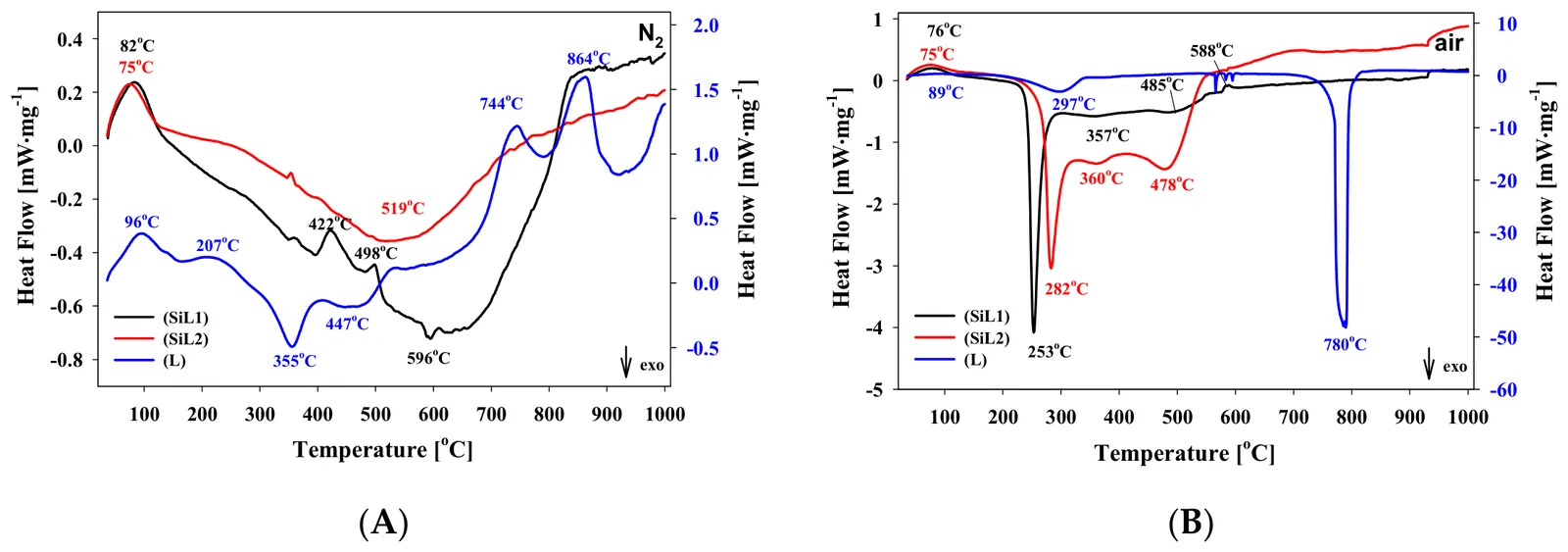
DSC curves of lignin and lignin-silica composites heated in nitrogen (**A**) and air (**B**).

Thermal analysis of lignin and composites was performed in nitrogen and synthetic air atmospheres, with simultaneous analysis of gaseous products using FTIR/MS methods. Figure 7A,B and Figure 8A,B present the thermogravimetric (TG/DTG) and DSC curves obtained from lignin decomposition. The pyrolysis process (Figure 7A and Figure 8A) is multistage and proceeds continuously across the entire temperature range up to 1000 °C [38,39,40,41,42]. The initial slight mass loss of approximately 3% at temperatures up to 150 °C results from an endothermic dehydration process, evidenced by characteristic bands in the MS peaks (Figure S2B, *m*/*z* 18) and FTIR spectra (Figure S3A, 3900–3400 cm^−1^). Further heating to 573 °C causes the greatest mass loss—approximately 31%—corresponding to a broad peak on the DTG curve with a minimum at 351 °C. This stage is exothermic, featuring two well-resolved peaks on the DSC curve at 355 °C and 467 °C. Notably, in the initial temperature range (160–210 °C), a glass transition was observed and determined to occur at 186 °C, a characteristic feature of this type of material [43]. According to literature data, the exothermic processes in this temperature range are primarily associated with the decomposition of side chains and aliphatic structures [38,39,40,42]. These mainly involve the cracking and reforming of carboxyl, carbonyl and ether groups, the decomposition of hydroxyl groups, and the breakdown of methoxy groups [41]. Analysis of the MS spectra (Figure S2, black line) revealed that the main gaseous pyrolysis products in this range were CO_2_ (*m*/*z* = 44), H_2_O (*m*/*z* = 18), and CH_4_ (*m*/*z* = 15), as indicated by intense peaks in the plots. These data were confirmed by IR spectra (Figure S3A). Bands at approximately 2359 and 669 cm^−1^ were assigned to CO_2_ stretching and bending vibrations, respectively. Signals in the 3700–3600 cm^−1^ range and around 1650 cm^−1^ are associated with the presence of water vapour. The band at approximately 3016 cm^−1^ was assigned to methane C–H stretching vibrations, while the signal around 1745–1761 cm^−1^ corresponds to C=O bond vibrations in carbonyl compounds and to the 2200–2000 cm^−1^ range in carbon monoxide. Additionally, MS analysis revealed small amounts of SO_2_ (*m*/*z* = 64). It should also be noted that formaldehyde and methanol may be among the products—as indicated by peaks at *m*/*z* 29, 30, and 31 (Figure S2C–E)—but since these signals are also characteristic of other compounds, it is difficult to confirm this conclusively. As the temperature rises, the decomposition processes slow down. Three mass-loss steps of 14%, 6%, and 5% were observed at 800, 906, and 1000 °C, respectively. These are endothermic processes. The first two are characterised by sharp peaks on the DTG and DSC curves, the minima of which are listed in Table 3. As a result of these reactions—which involve the breaking of strong bonds in aromatic rings and the gradual formation of carbonaceous materials—it was determined that CO_2_ and CO were the main products throughout this range, while water was also evolved up to a temperature of 900 °C (Figures S2 and S3). Our studies did not confirm the emission of aromatic compounds during the pyrolysis of the lignin under investigation (or detected only negligible amounts), even though such products are frequently described in the literature [43,44,45,46].

Studies on the thermal stability of lignin in synthetic air (Figure 7B and Figure 8B) revealed a decomposition pattern up to 600 °C that was broadly similar to the previously described pyrolysis process. In this case, two stages can also be distinguished within this range. The first stage, extending up to 150 °C with a mass loss of approximately 3%, is an endothermic process associated with dehydration (Figure S2B). When the temperature was raised to approximately 620 °C, the sample mass decreased by 33% due to an exothermic process; two peaks were recorded on the DTG curves at 296 and 359 °C, with the intensity of the first process being significantly higher. As in the case of pyrolysis, the post-reaction gases were found to contain primarily CO_2_ (*m*/*z* = 44), H_2_O (*m*/*z* = 18), and CH_4_ (*m*/*z* = 15), along with small amounts of SO_2_ (*m*/*z* = 64) (Figure S2).

Figure 7C and Figure 8A show the thermogravimetric and DSC curves for the thermal decomposition of the obtained SiL1 (black line) and SiL2 (red line) composites. In both cases, three main stages of pyrolysis can be distinguished. The first stage, involving a slight mass loss of approximately 2%, is associated with the endothermic process of water removal, as evidenced by a distinct peak in the MS spectrum (Figure S4B). The most significant mass losses—14% for sample SiL1 and 16% for sample SiL2—are observed in the 150–620 °C temperature range. This transformation occurs virtually continuously throughout the range and is accompanied by broad peaks with minima at 409 °C (SiL2) and 446 °C (SiL1), although minor effects can be observed for sample SiL1 at lower temperatures, down to 320 °C. In this range, a clear shift in the DTG peaks towards lower temperatures is visible for sample SiL2, likely due to the higher lignin content used in the synthesis. This also indicates lower thermal stability for this material. The processes occurring during the main stage are primarily exothermic, although two endothermic events at 422 °C and 498 °C can be observed for sample SiL1, which may indicate the dehydroxylation of surface silanol groups on the silica. Analysis of the MS spectra of the gaseous products (Figure S4) indicates that CO_2_ is the main decomposition product in this range, resulting from carbonisation and the decomposition of lignin surface groups. The intensity of this process was significantly higher for the SiL2 sample (containing a larger amount of modifier), and it occurred in a single stage (Figure S4F, blue line). For the SiL1 sample, the process proceeded in two stages, as evidenced by peaks in the 400–600 °C temperature range. Additionally, the release of H_2_O (Figure S4B) and CH_4_ (Figure S4A) was observed. Infrared analysis also revealed the release of carbon monoxide (Figure S3C,E; 2175/2116 cm^−1^). Analysis of the MS and FTIR spectra also showed signals indicating the presence of numerous other volatile organic products containing aliphatic groups and carbonyl compounds (1745/1761 cm^−1^); this was partly due to the synthesis method, which did not include a calcination step. Because of uncertainties in interpretation, no detailed analysis of these signals was performed. The mass loss in the 600–1000 °C was more than twice as high for the SiL2 sample (Table 3, Δm = 7%), which had a higher lignin content compared to the SiL1 sample (Δm = 2.6%). The processes in this range—which were primarily endothermic—were accompanied only by the emission of carbon dioxide (Figure S3C,E).

In a synthetic air atmosphere, decomposition of the composites proceeds in three stages (Figure 7D and Figure 8B). The first stage, occurring up to 126 °C with a minor mass loss of less than 2%, is associated with dehydration. The greatest mass loss is observed in the 150–600 °C temperature range. The phenomena occurring during this decomposition step are markedly discontinuous, allowing two main stages to be distinguished. The first stage—extending up to approximately 330 °C, with mass losses of about 8 and 10% for SiL1 and SiL2, respectively—is very rapid and accompanied by a sharp peak on the DTG curve (Figure 7D) associated with heat release (Figure 7B). For sample SiL1, the temperature of this stage was 30 °C lower than for the sample with the higher lignin content. At higher temperatures within this range, the energetic effects were considerably less intense, and peaks were observed on the DSC curves at approximately 360 and 480 °C. Total mass losses in this range (300–560 °C) amounted to 9% (SiL1) and 15% (SiL2) (Table 3, Figure 7D). Analysis of the gaseous products revealed that the first event—which was endothermic—resulted in the release of CO_2_ (Figure S4F), H_2_O (Figure S4B) and CH_4_ (Figure S4A), along with small amounts of CO (Figure S3D,F), whereas the second stage (up to 600 °C) involved only carbon dioxide and a small amount of water.

These effects were primarily associated with carbonisation, calcination, and the oxidation of organic carbon in the resulting carbon structures, as well as transformations within the silica. For sample SiL1, the energetic effect observed at 588 °C is associated with a polymorphic transformation of silica. At higher temperatures, up to 1000 °C, slight mass losses of approximately 2% are attributed to the oxidation of residual carbonaceous materials.

Under a synthetic air atmosphere, the total mass loss at 1000 °C for pure lignin, SiL1, and SiL2, corrected for mass loss due to dehydration, was used to estimate the organic phase content of the composites—25 wt% for SiL1 and 38 wt% for SiL2. This indicates that the organic phase content of SiL2 is approximately 1.5 times that of SiL1.

### 2.3. Adsorption Equilibrium and Kinetics of Triarylmethane Dyes on Silica–Lignin Composites

To determine the adsorption capacities and rates for the systems: dyes—silica–lignin composites, adsorption isotherm and kinetic measurements were carried out. Triarylmethane dyes crystal violet (CV), basic fuchsin (BF), and malachite green (MG) were selected as adsorbates. The physicochemical properties of applied dyes are summarized in Table 4.

**Figure 9:**
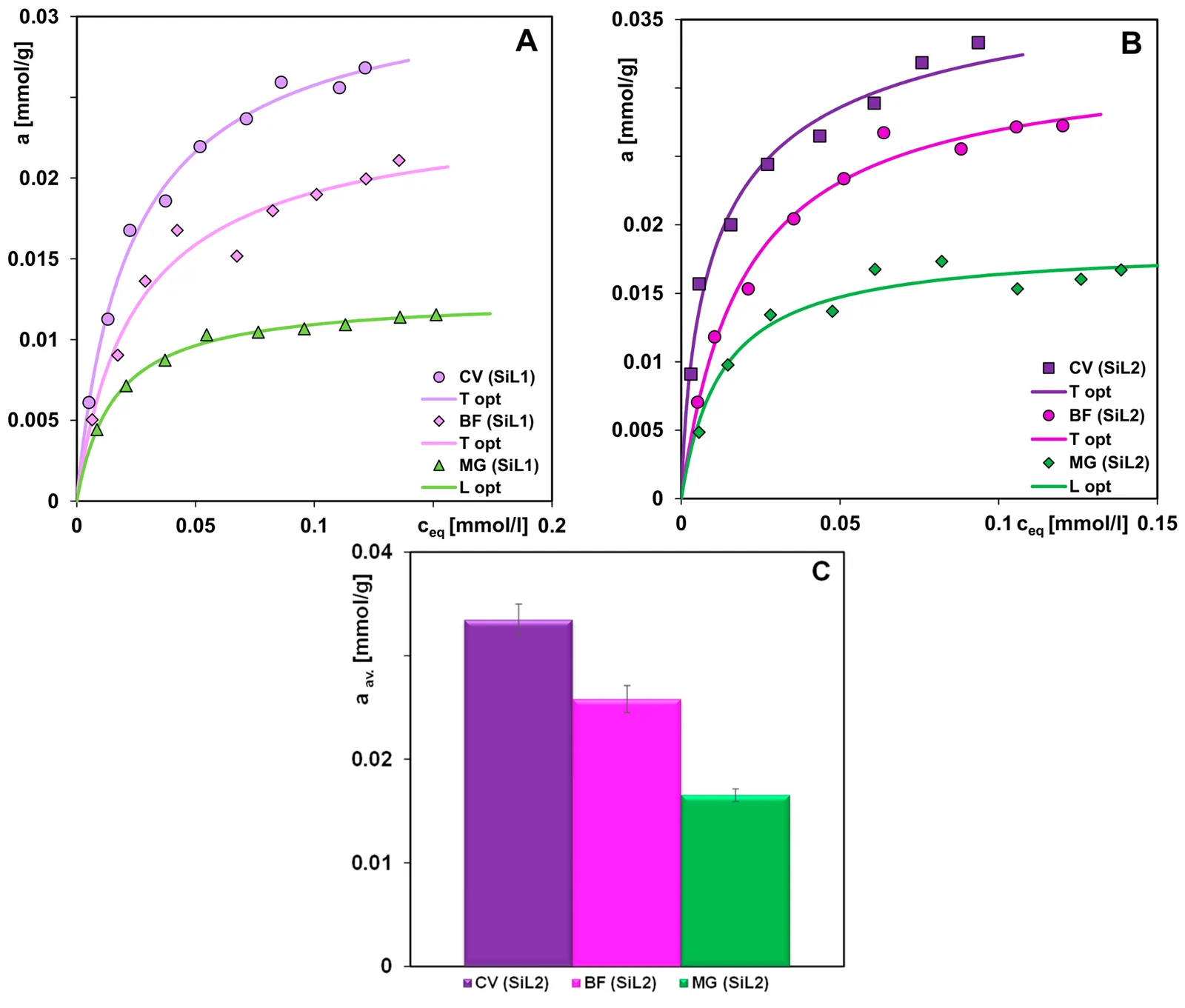
Adsorption isotherms of various dyes on silica–lignin composites SiL1 (**A**) and SiL2 (**B**). The mean amounts of dyes adsorbed onto SiL2, calculated from three independent experimental trials, along with the corresponding standard deviations (m = 0.05 g; c_0_ = 0.177 mmol/L; V = 25 mL, T = 25 °C, R = 120 rpm) (**C**).

Figure 9A,B present the adsorption isotherms of the investigated dyes on the SiL1 and SiL2 composites. For both adsorbents, a similar trend in adsorption efficiency was observed. The adsorption capacity of the composites toward the investigated dyes decreased in the following order: CV > BF > MG, while SiL2 exhibited a better adsorption performance than SiL1. This relationship was confirmed by analysis of the adsorption capacity parameter (a_m_), determined using the Generalized Langmuir equation (GL). For SiL1, the a_m_ values for CV, BF, and MG were 0.033, 0.025, and 0.013 mmol/g, respectively, whereas for SiL2 they were 0.041, 0.032, and 0.018 mmol/g, respectively.

The batch consistency for crystal violet, basic fuchsin, and malachite green adsorption on the selected silica–lignin composite (SiL2) was evaluated based on three independent trials conducted under identical experimental conditions: m = 0.05 g; c_0_ = 0.177 mmol/L; V = 25 mL, T = 25 °C, R = 120 rpm (Figure 9C). The standard deviations ranged from 3.6% to 5.0%, indicating low variability among the measurements and good repeatability of the adsorption process.

Under the experimental conditions, all investigated adsorbates were present as monovalent cations, whereas the composite surfaces exhibited a negative charge, as confirmed by potentiometric titration measurements. This surface charge can be attributed to the partial deprotonation of silanol groups in the silica matrix, as well as carboxyl, phenolic, and hydroxyl groups derived from the lignin component. These surface characteristics suggest that electrostatic interactions make a substantial contribution to the adsorption process. At the same time, the lignin structure is rich in aromatic moieties that may participate in π–π interactions with the aromatic systems of the dyes. The coexistence of these interaction mechanisms may account for the higher adsorption efficiency of SiL2, which contains a higher proportion of lignin. The textural properties of SiL2 may also favor adsorption, as this composite exhibited a higher contribution of mesopores to the total pore volume (V_p_/V_t_ = 0.92) than SiL1 (V_p_/V_t_ = 0.76). Owing to the relatively large size of the adsorbate molecules, some micropores may be inaccessible because of molecular sieving. Consequently, dye molecules are expected to preferentially occupy mesopores, whose contribution to the porous structure is particularly high in SiL2.

The structural characteristics of the dyes may further differentiate the behavior of the individual adsorption systems. The structure of crystal violet, characterized by the presence of three dimethylamino groups, results in relatively high hydrophobicity compared with the other dyes. This feature may promote interactions with hydrophobic fragments of lignin, including methoxy groups, as well as π–π interactions between aromatic systems. Moreover, the distribution of the dimethylamino groups within the extended aromatic system imparts a certain degree of molecular symmetry, which may favor the ordered organization of the molecules on the adsorbent surface, including the formation of molecular associates [47,48]. In the case of malachite green, for which the lowest adsorption capacity was observed, differences in the structure and distribution of substituents relative to crystal violet may be of particular importance. One of the aromatic rings lacks a dimethylamino substituent, thereby affecting both molecular symmetry and electron-density distribution. Consequently, MG may have a lower tendency to form ordered associates on the adsorbent surface. Differences in the geometry of the two cations may also affect their adsorption behavior, as molecular geometry determines their orientation relative to the negatively charged active sites of the composites.

**Figure 10:**
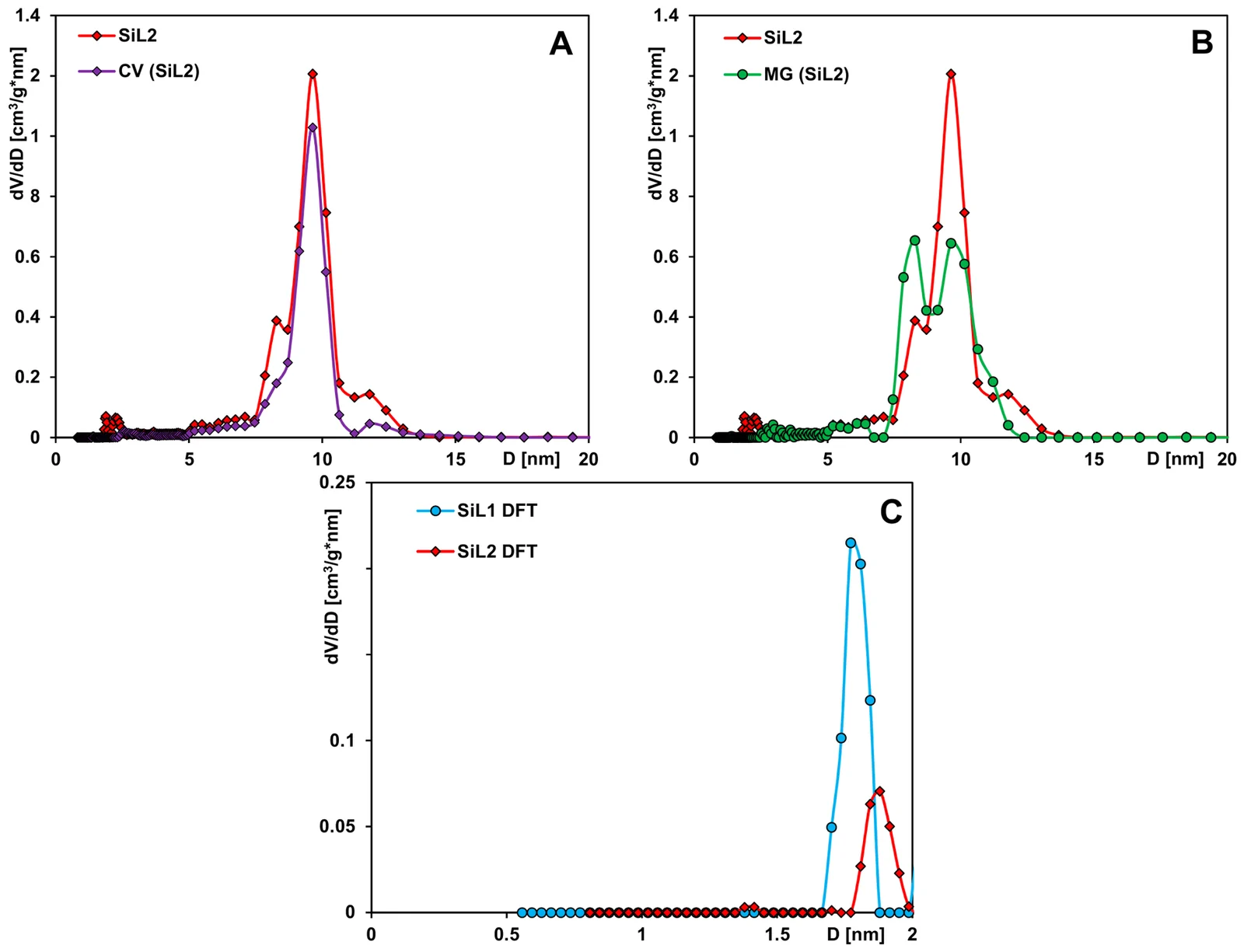
Pore size distributions based on DFT calculations for the samples measured after the adsorption process (**A**,**B**). The micropore size distributions of the initial composites (**C**).

Differences in the adsorption behavior of crystal violet and malachite green are reflected in the pore size distributions obtained after the adsorption process (Figure 10A,B). The CV(SiL2) sample exhibits a relatively homogeneous pore size distribution, indicative of a more ordered arrangement of adsorbed molecules within the pore structure. In contrast, the MG(SiL2) sample shows a distinct bimodal distribution, suggesting heterogeneous pore filling and a less ordered organization of the adsorption layer. This observation points to differences in the packing efficiency and intermolecular interactions of the dyes within the porous adsorbent matrix.

Analysis of the curves (Figure 10A,B) in the range of 0–2 nm reveals that the SiL2 composite contains a population of micropores that is not detectable after dye adsorption. These changes may result from either the volumetric filling of micropores by dye molecules, molecular sieving or the blocking of pores whose dimensions are smaller than those of the adsorbate molecules. In the latter cases, although the pores are not occupied by dye molecules, nitrogen molecules cannot penetrate them during textural analysis. A comparison of the micropore size distributions of the two initial composites (Figure 10C) indicates that the micropores in SiL1 are shifted towards smaller sizes. This suggests that molecular sieving or pore blocking are more likely to occur during dye adsorption on SiL1, rendering some pores inaccessible to the dye molecules.

BF, characterized by the smallest molecular size among the investigated dyes, was removed from the aqueous phase with intermediate efficiency. Electrostatic interactions likely remained the primary mechanism responsible for its binding, while its relatively small molecular size may have facilitated denser packing on the adsorbent surface and improved access to some pores. However, the presence of three hydrophilic amino groups may have reduced the contribution of hydrophobic and dispersive π–π interactions compared with CV. Hydrogen bonding and donor–acceptor interactions between functional groups of the dye and active sites on the composite surfaces may, in turn, have played an important role.

As mentioned above, the Generalized Langmuir equation (GL) was applied to describe the experimental adsorption isotherms (Table 5). The adsorption of crystal violet and basic fuchsin was best described by the Toth equation (T), whereas the Langmuir equation (L) provided the best fit for malachite green. For three adsorption systems, the heterogeneity parameter *n* ranged from 0.62 to 0.91 at *m* = 1, suggesting a heterogeneous and asymmetric distribution of adsorption energies with broadening toward lower energies. For the remaining systems, *n* ranged from 0.98 to 1, reflecting a more energetically homogeneous surface sites with respect to interactions with the respective adsorbate. Given the observed heterogeneity of the resulting composite materials—which clearly feature two distinct phases with differing properties (silica and lignin)—one would expect significant energetic heterogeneity in the adsorption systems under study. However, the results suggest that most dye-composite systems exhibit negligible or slight heterogeneity. This effect can be attributed to the compensation of energetic heterogeneity by interactions within the adsorption phase. In reality, the systems exhibit an overall non-ideality resulting from a combination of factors: energetic heterogeneity, specific and dispersive interactions, and electrostatic interactions. Thus, the mutual compensation of these various factors may account for the slight overall deviation from homogeneity observed in the systems studied. [49,50]. Analysis of the equilibrium constant values (log *K*), which ranged from 1.63 to 1.80 for the SiL1 systems and from 1.72 to 2.27 for the SiL2 systems, revealed a higher affinity of the dyes for the composite containing a greater proportion of lignin and characterized by a higher contribution of mesopores. The higher log *K* values obtained for the SiL2 systems further demonstrate stronger adsorbate—adsorbent interactions in energetic terms.

FTIR correlation maps of the silica–lignin composite after adsorption of cationic dyes (crystal violet (CV), basic fuchsin (BF), and malachite green (MG)) enable assessment of the spatial distribution of the adsorbates and their association with the individual composite phases.

**Figure 11:**
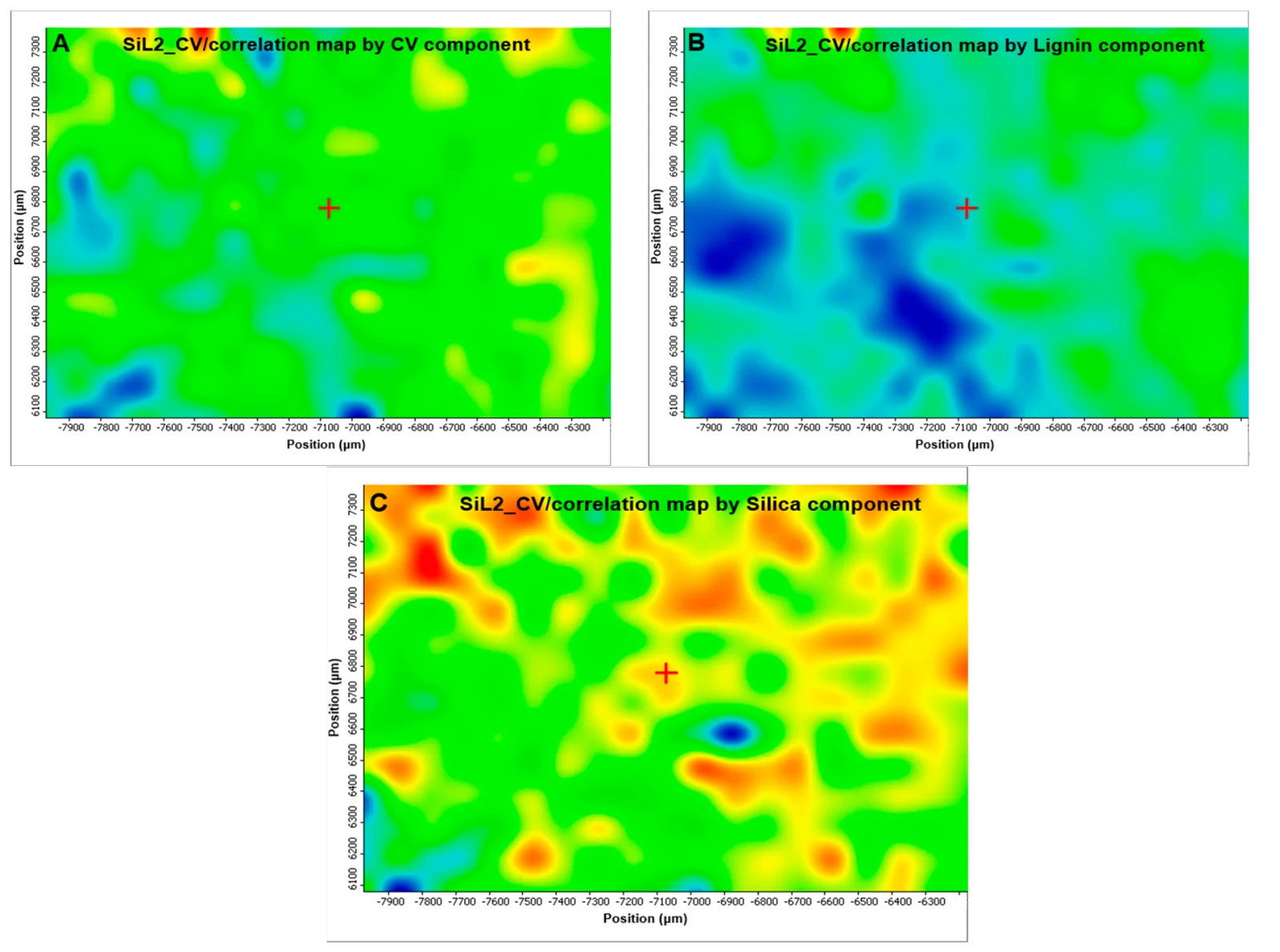
FTIR correlation maps of the silica–lignin composite SiL2 with crystal violet (**A**–**C**): (**A**) mapping based on the dye component, (**B**) based on lignin, (**C**) based on silica.

For the CV-loaded composite (Figure 11), the correlation map referenced to the dye band (Figure 11A) indicates a largely uniform surface distribution of CV, extending across both lignin-enriched regions (Figure 11B) and silica-enriched regions (Figure 11C). The lack of dye-depleted domains implies that the composite provides a continuous, functionally active adsorption interface that supports formation of an adsorbed CV layer. The correlation maps referenced to lignin (Figure 11B) and silica (Figure 11C) further show that zones of maximal signal intensity for both components are spatially coincident and, concurrently, correspond to the regions exhibiting the highest CV uptake. This spatial co-localization suggests that lignin and silica do not behave as separate, mutually competing phases; instead, they operate cooperatively, whereby lignin contributes aromatic and polar functionalities capable of π–π interactions, hydrogen bonding, and electrostatic attraction, while silica supplies a silanol-rich surface able to participate in an extended interaction network. Such complementarity is consistent with multipoint anchoring of CV at sites associated with both phases. Moreover, the comparatively regular molecular geometry of CV is expected to facilitate ordered surface organization and efficient packing through geometric matching to available binding motifs on lignin and silica. This interpretation is consistent with the adsorption isotherm results, which indicate that CV achieves the highest adsorption capacity among the dyes examined (relative to BF and MG).

**Figure 12:**
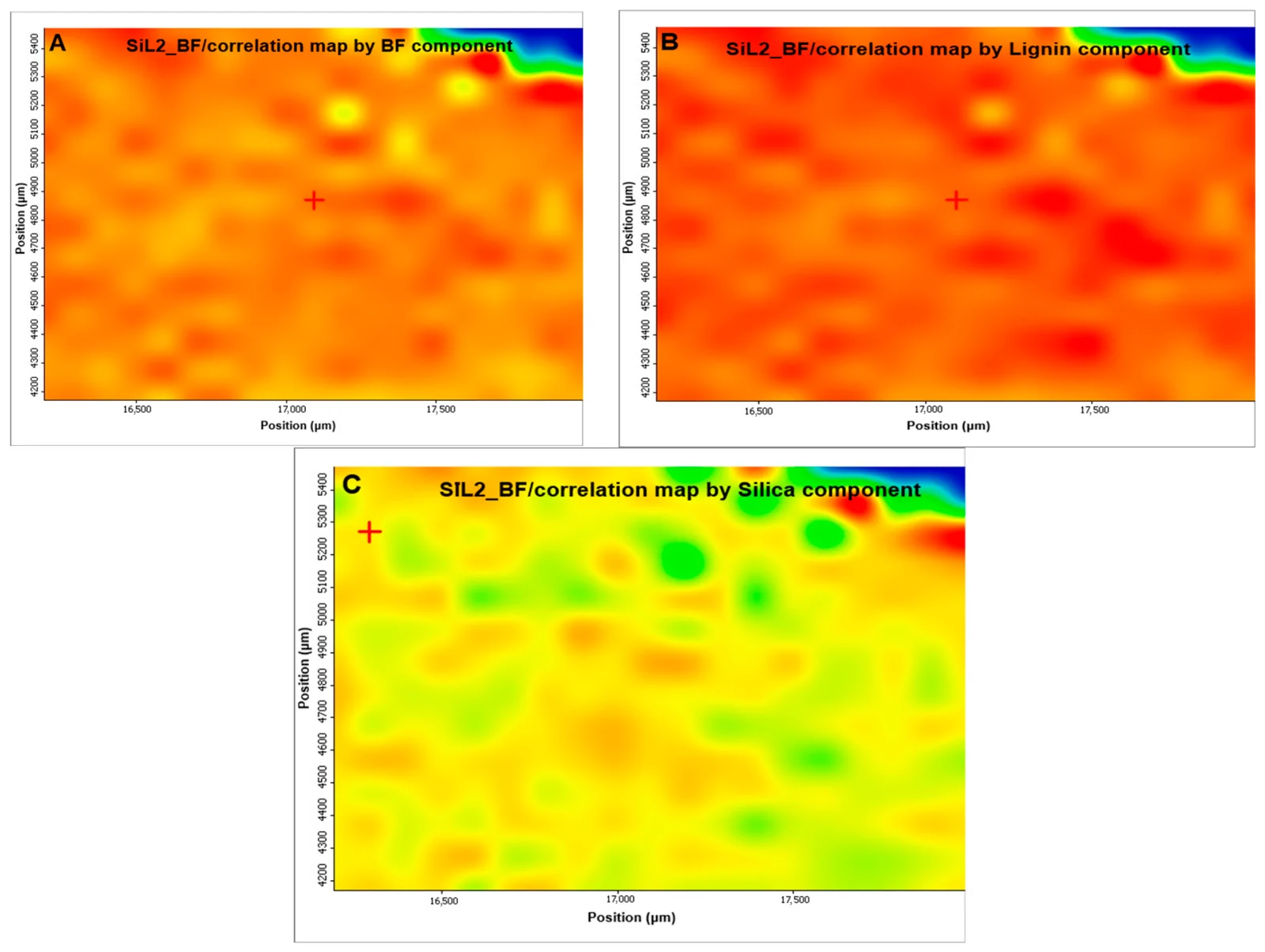
FTIR correlation maps of the silica–lignin composite SiL2 with BF dye (**A**–**C**): (**A**) mapping relative to the dye component, (**B**) relative to lignin, (**C**) relative to silica.

FTIR correlation maps of the silica–lignin composite after adsorption of basic fuchsin (BF) demonstrate a dye localization pattern that is clearly distinct from that observed for crystal violet (CV) (Figure 12). In contrast to CV, whose spectral contribution is homogeneously distributed and exhibits strong spatial correlation with both lignin and silica, BF displays a pronounced preferential association with lignin-enriched domains. The correlation map referenced to the BF component (Figure 12A) indicates that BF intensity co-localizes predominantly with regions characterized by elevated lignin content (Figure 12B). This marked spatial concordance supports the conclusion that lignin constitutes the primary adsorption phase for BF. Conversely, the spatial correlation between BF and silica is weak (Figure 12C): silica-rich regions do not coincide with high BF signal intensity, implying limited access of BF to, or retention within, the porous silica network. Although porous silica would be expected to facilitate adsorption for dye molecules with appropriate size, geometry, and capacity for organized packing within an adsorbed layer, BF does not appear to satisfy these criteria under the present conditions. Basic fuchsin (BF), owing to its comparatively irregular molecular geometry and increased steric bulk, exhibits a reduced capacity to self-organize on the adsorbent surface. The limited structural regularity of BF impairs its conformational accommodation within the porous silica matrix, resulting in lower adsorption in this phase and a preferential association of BF molecules with lignin domains. Consequently, BF predominantly forms a disordered surface-adsorbed layer with minimal diffusion or penetration into the interior of the composite structure.

**Figure 13:**
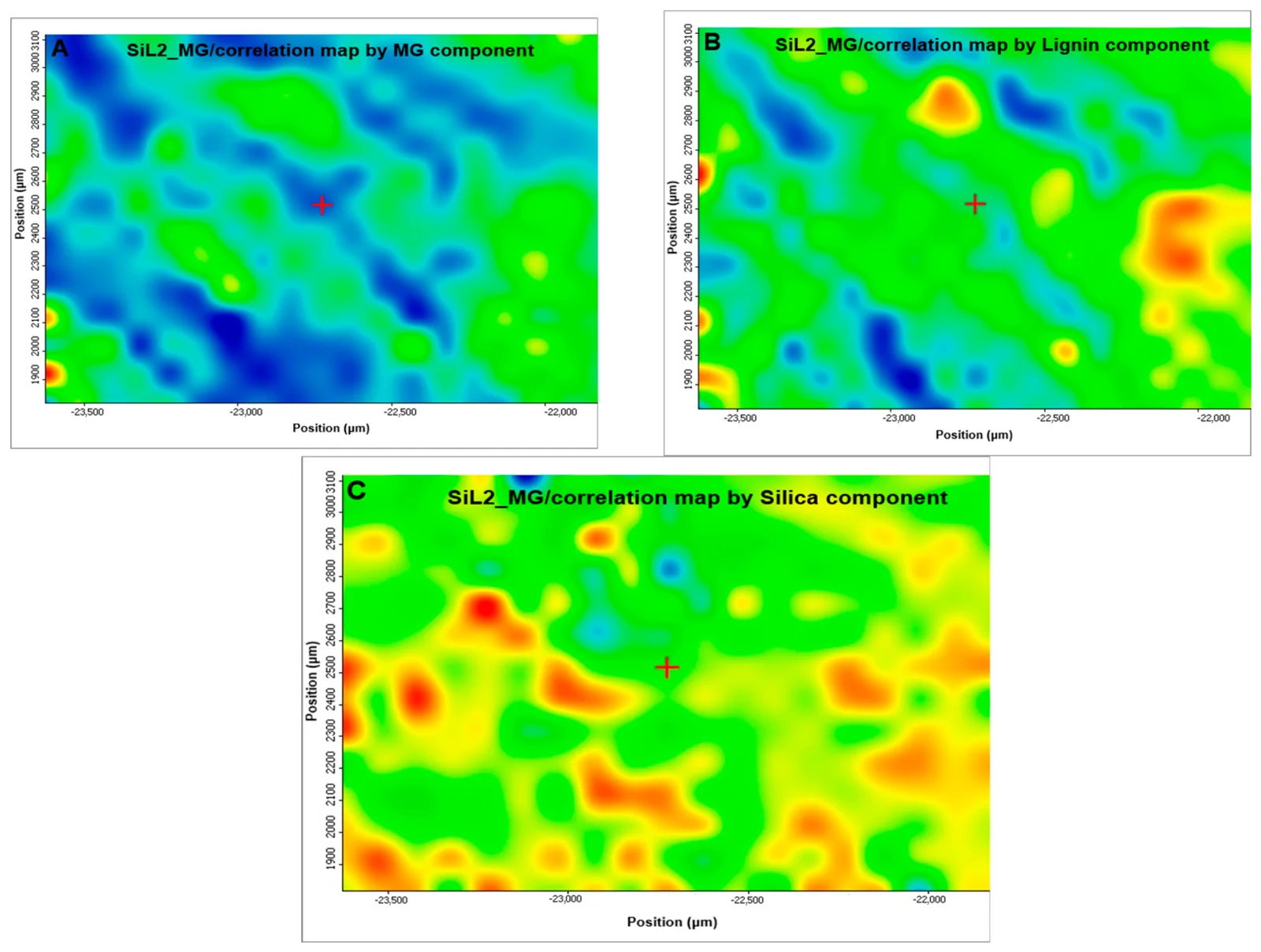
FTIR correlation maps of the silica–lignin composite SiL2 with MG dye (**A**–**C**): (**A**) mapping relative to the dye component, (**B**) relative to lignin, (**C**) relative to silica.

The minimal adsorption capacity observed for malachite green is consistent with the trends inferred from the FTIR correlation maps (Figure 13). Specifically, the dye exhibits only moderate correlation with lignin and a negligible correlation with silica, suggesting suboptimal engagement of both the available surface area and the composite’s porous architecture. In contrast to CV, whose more regular molecular geometry is conducive to a more ordered arrangement within the adsorbed layer, MG appears to be less compatible, both structurally and electrostatically, with the hybrid adsorbent. This reduced complementarity plausibly accounts for its lowest adsorption performance among the dyes evaluated.

Analysis of FTIR maps reveals varied interactions between the studied dyes and the organic and inorganic phases of the composites: crystal violet (CV) interacts effectively with both phases; basic fuchsin (BF) shows a greater affinity for the organic phase while also adsorbing onto the silica; and malachite green (MG) adsorbs exclusively onto the lignin phase. This effect is corroborated by adsorption studies conducted for all the investigated adsorption systems. An analysis of the ratios of adsorption capacities, *a_m(SiL2/SiL1)_*, shows an increase in the order: CV < BF < MG: *a_m,CV(SiL2/SiL1)_* = 1.24, a*_m,BF(SiL2/SiL1)_* = 1.28, a*_m,MG(SiL2/SiL1)_* = 1.38. The highest value of this ratio for malachite green indicates the strongest correlation between the amount of adsorbed dye and the content of the organic phase in the case of MG. These results highlight the importance of adsorbent composition, particularly the lignin content, and its influence on development of the adsorption capability of the materials.

On the basis of the FTIR spectra, the vibrational features of amorphous silica and alkaline lignin are readily discernible (Figure S5A; black and blue curves respectively). For lignin, the absorptions at 1594 and 1513 cm^−1^ constitute clear evidence of an aromatic backbone. Bands observed at 1265 and 1031 cm^−1^ are attributable to oxygenated functionalities, consistent with ether linkages and phenolic groups, whereas the broad band centered at 3420 cm^−1^ reflects a high abundance of hydroxyl groups, which underpin lignin’s propensity for hydrogen-bond formation. The pronounced intensity of the aromatic vibrations further suggests that the phenylpropane units characteristic of alkaline lignin remain largely preserved. For the silica reference, the strong bands at 1087, 800, and 463 cm^−1^ confirm the presence of a typical amorphous silica network based on SiO_4_ tetrahedral units. The absorption at 961 cm^−1^ indicates a substantial population of silanol (Si–OH) groups, which may serve as reactive or interaction sites for lignin attachment. In addition, the broad O–H stretching band at 3451 cm^−1^ together with the signal at 1640 cm^−1^ are consistent with adsorbed water associated with the high specific surface area of the silica.

Relative to pristine silica, the FTIR spectra of the composites (green and orange traces (Figure S5A) corresponding to SiL1 and SiL2, respectively) exhibit additional absorption features attributable to lignin. Notably, the band centered at approximately 2930 cm^−1^ is intensified and broadened, consistent with contributions from aliphatic C–H stretching vibrations of lignin. In addition, a weak feature in the 1384–1450 cm^−1^ range, assignable to aromatic skeletal vibrations and CH_3_/CH_2_ deformation modes, is more clearly resolved in sample K2, in accordance with its higher lignin loading. Collectively, these spectral modifications substantiate successful lignin incorporation within the silica matrix, while the diagnostic bands of amorphous SiO_2_ remain preserved.

Based on the FTIR spectra presented in Figure S5B, it can be observed that after adsorption of all three dyes, the basic structure of the SiL2 composite remains preserved. This is evidenced by the presence of characteristic silica bands at ca. 1080 cm^−1^ (νas Si–O–Si), 960 cm^−1^ (Si–OH), 800 cm^−1^ (νs Si–O–Si), and 460 cm^−1^ (δ O–Si–O), whose positions do not change significantly. This indicates that the adsorption process does not lead to reconstruction of the silica framework of the adsorbent. The most noticeable differences between the pristine SiL2 sample and the materials after adsorption occur in the 1900–1500 cm^−1^ region, which includes bands associated with hydroxyl groups and adsorbed water (ca. 1637 cm^−1^), as well as aromatic vibrations originating from lignin. The intensity and shape of signals in this region change in accordance with the adsorption efficiency of the individual dyes. The greatest deviation from the initial spectrum is observed for the SiL2_CV sample. The spectrum after crystal violet adsorption shows the most pronounced changes in the 1900–1500 cm^−1^ range, indicating strong interactions between dye molecules and oxygen-containing groups present on the composite surface. This result is fully consistent with the highest adsorption capacity obtained for CV. Marked changes in the band around 1637 cm^−1^ suggest the involvement of lignin hydroxyl groups and silica silanols in the dye-binding process. The SiL2_BF sample shows intermediate changes. Modifications of bands in the 1900–1500 cm^−1^ range are visible, but their intensity is lower than for CV. This corresponds to the intermediate adsorption capacity of the composite toward basic fuchsin and indicates a similar, yet less intense, interaction mechanism with surface oxygen groups of the adsorbent. The smallest differences relative to the pristine sample are observed for SiL2_MG. The spectrum after malachite green adsorption is the most similar to that of the adsorbent before adsorption, and the changes in the 1900–1500 cm^−1^ region are the least pronounced. This correlates with the lowest adsorption efficiency for this dye and indicates weaker interactions with surface active sites. Additionally, for all samples after adsorption, changes in intensity and slight broadening of the band in the 2925–2930 cm^−1^ region, corresponding to C–H stretching vibrations, can be observed. This effect is related to the presence of adsorbed dye molecules containing substituted aromatic rings and alkyl groups. Overall, the FTIR analysis indicates that adsorption of the investigated dyes occurs mainly via surface hydroxyl groups of lignin and silanol (Si–OH) groups of silica, leading to electrostatic interactions and hydrogen bonding. The extent of changes observed in the FTIR spectra is consistent with the order of adsorption capacity: CV > BF > MG, providing additional confirmation of the proposed adsorption mechanism.

The equilibrium adsorption studies of cationic dyes on the silica–lignin composites were complemented by kinetic analysis. Exemplary kinetic data in the form of absorption spectra are presented in Figure S6A in the Supplementary Materials. Figure S6B shows the spectra of the same dye recorded during contact with native lignin. In this case, a distinct change in the spectral profile was observed, manifested as a shift in the absorption maxima toward longer wavelengths in the visible radiation region. This effect may be attributed to a change in the molecular form of the adsorbate. Simultaneously, a substantial increase in the signal intensity in the shorter-wavelength region was observed, resulting from partial dissolution of lignin. An analogous phenomenon was observed during contact between lignin and the other dyes. The absence of this effect in the dye/composite systems supports the effective immobilization of the lignin component within the silica matrix and indicates that no appreciable lignin release occurred under the conditions of the adsorption experiments.

**Figure 14:**
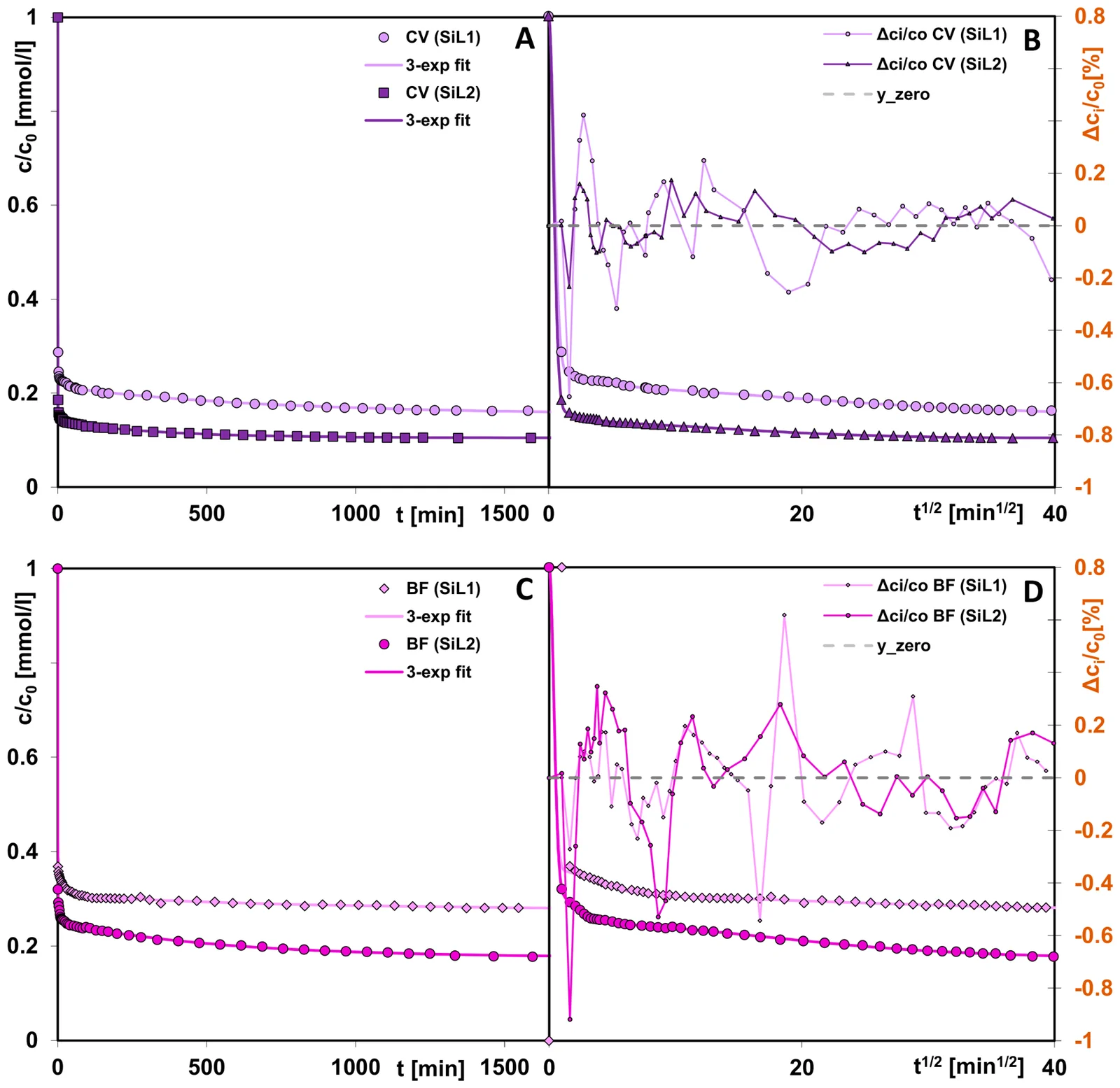

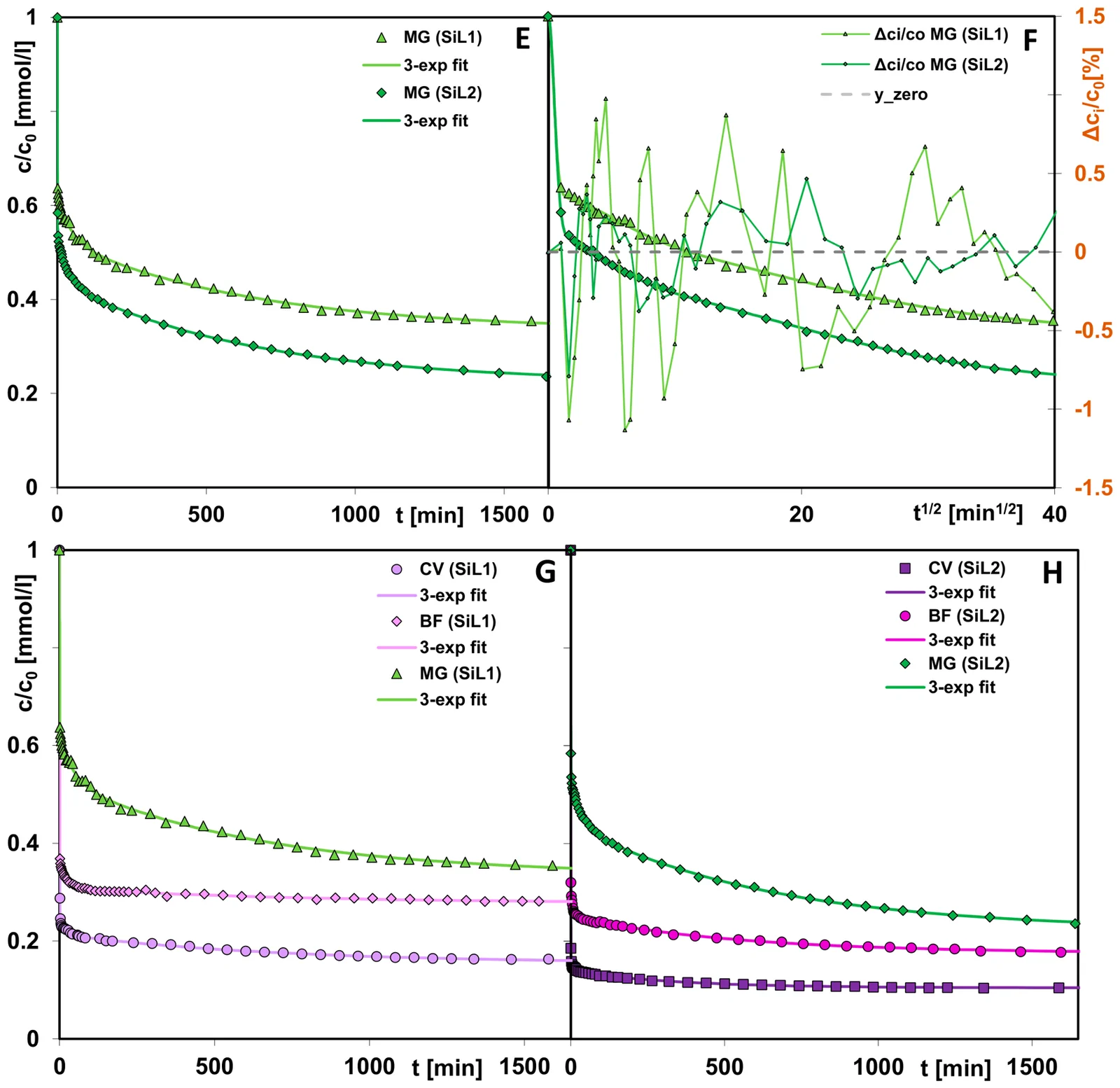
Comparison of the adsorption kinetics of CV (**A**,**B**), BF (**C**,**D**) and MG (**E**,**F**) on the silica–lignin composites at the coordinates: relative concentration ~ time (**A**,**C**,**E**) and concentration ~ square root of time (**B**,**D**,**F**). Comparison of the adsorption kinetics of various dyes on composites SiL1 (**G**) and SiL2 (**H**). The lines correspond to the fitted m-exponential equation.

The kinetic curves for the investigated systems are presented as relative concentration versus time and relative concentration versus the square root of time (Figure 14). Analysis of the adsorption kinetics as a function of composite type (Figure 14A–F) revealed similar curve profiles, while differences in adsorption efficiency persisted throughout the experiment. These differences can be attributed primarily to variations in the contribution of individual types of active sites and their accessibility to dye molecules. The higher lignin content of SiL2, together with the greater availability of active sites associated with its higher mesopore contribution, resulted in higher adsorption efficiency at the individual stages of the process. For the systems containing basic fuchsin, differentiation of the kinetic curves was observed after approximately 200 min. At this point, adsorption on SiL1 approached a state of dynamic equilibrium, whereas adsorption on SiL2 continued, although at a progressively lower rate, until the end of the measurement period. The relatively small size of the basic fuchsin molecule facilitated its rapid transport to the available adsorption sites, which, in the case of SiL1, resulted in relatively rapid saturation. The different quantitative composition of SiL2, characterized by a higher lignin content and, consequently, a greater number of potential adsorption sites associated with lignin, delayed the attainment of equilibrium.

More pronounced differences in adsorption kinetics were observed as a function of dye type. The kinetic curves obtained for crystal violet and basic fuchsin were similar and characterized by a very rapid initial course, whereas adsorption of malachite green proceeded considerably more slowly. A quantitative comparison of the adsorption rates was performed using the *t*_50%_ parameter, defined as the time required to achieve a 50% reduction in dye concentration relative to its initial value. This parameter, together with the other kinetic parameters discussed below, was determined by fitting the experimental data with a multiexponential equation (m-exp). The *t*_50%_ values for CV and BF were below 1 min, whereas those for MG ranged from 13 to 125 min, depending on the composite used. The observed differences in adsorption kinetics cannot be explained solely by molecular size. Despite having the largest molecules among the investigated dyes, CV was adsorbed both very rapidly and in the greatest amount. In addition to molecular size, the adsorption rate is governed by several factors, including adsorbate affinity for the adsorbent surface, the nature of adsorbate—adsorbent interactions, and the ability of the molecules to access adsorption sites within the porous structure. In the case of CV, its high adsorption rate and capacity may be related to favorable interactions between the dye molecules and the surfaces of the composites. Stronger intermolecular interactions provide the driving force for faster adsorption. The structural characteristics of CV may also be relevant, particularly its relatively regular molecular geometry, which may facilitate favorable orientation relative to the adsorbent surface and organization within the adsorbed layer. Its tendency to form associates on the solid surface may have further contributed to more efficient utilization of the available surface area and greater adsorption rate. For BF, the smaller molecular size may facilitate faster transport through the bulk solution and interfacial region, as well as rapid occupation of adsorption-active sites. This may account for the rapid kinetics of the process, although the final amount of bound dye was lower than that observed for CV. The slowest adsorption and lowest affinity for the composites were observed for MG. These differences may result from the distinct molecular structure and geometry of MG, as well as from the nature of its interactions with the adsorbent surface. The asymmetric structure of MG may limit the possibility of favorable molecular orientation relative to the surface and thereby reduce the extent to which the available adsorption sites are utilized. Its lower tendency to form ordered structures on the solid surface may also contribute to the lower overall adsorption capacity and slower adsorption rate.

Analysis of the rate constant (log *k_avg_*) and half-time (*t*_0.5 avg_) determined for the investigated systems revealed some discrepancies between these parameters and the kinetic efficiency assessed from the overall shape of the kinetic curves. The relatively narrow ranges of log *k_avg_* (−0.07–0.55) and *t*_0.5 avg_ (0.20–0.82) suggest relatively small differences among the analyzed systems, whereas direct analysis of the kinetic curves clearly indicates slower adsorption of MG. It should be emphasized that these parameters describe the amount of dye adsorbed relative to the amount bound under equilibrium or near-equilibrium conditions. In contrast, the total dye uptake, expressed by the *u_eq_* parameter, varied considerably and amounted to 0.85–0.90 for CV, 0.72–0.82 for BF, and 0.67–0.78 for MG. Furthermore, a substantial decrease in relative concentration, ranging from 0.17 to 0.65, was already observed within the first minute of the process. Therefore, direct comparison of *k_avg_* and *t*_0.5 avg_ values is most appropriate for systems characterized by similar *u_eq_* values, which was not the case for the systems investigated here.

**Figure 15:**
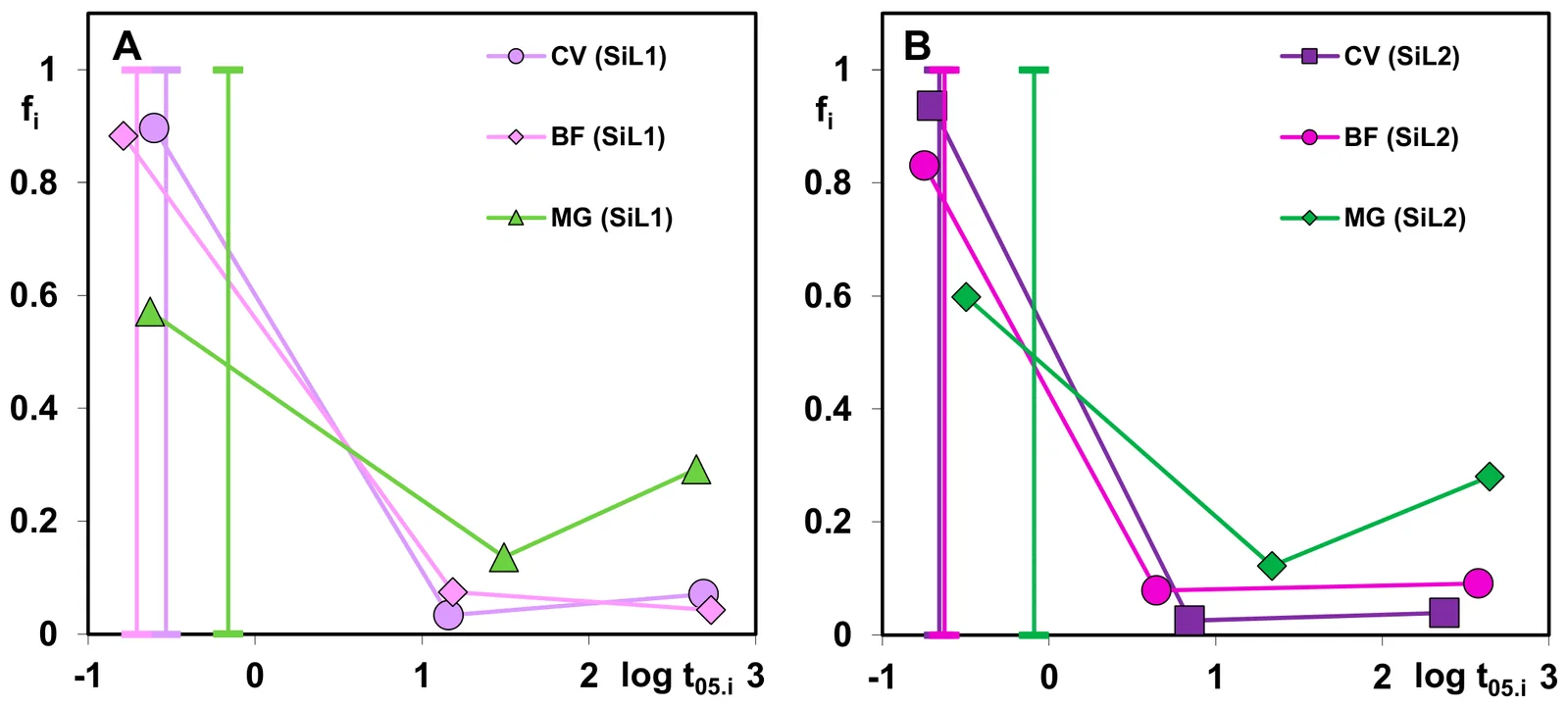
Distribution of half-time t_0.5,i_ for dyes’ adsorption on the composites SiL1 (**A**) and SiL2 (**B**).

The differences in the adsorption behavior of dyes on the composites are well reflected by the distributions of the half-times *t*_0.5,*i*_ assigned to the individual terms of the multiexponential equation (Figure 15). Systems characterized by a greater contribution of shorter half-times exhibited higher adsorption rate. For CV and BF, the fastest initial stage of adsorption accounted for the removal of 83–94% of the dye from the solution, the intermediate stage for 2–8%, and the slowest stage for a further 4–9% removal. For MG, these contributions were more evenly distributed, amounting to 57–60%, 12–14%, and 28–29%, respectively. Regardless of the composite type, the shape of distributions obtained for CV and BF were asymmetric and shifted toward a high contribution of short half-times. In contrast, the distributions for MG were more symmetrical and flattened, indicating a more uniform contribution of the half-times *t*_0.5,*i*_ values to the overall adsorption process.

The adsorption of solutes onto porous adsorbents generally proceeds through three consecutive stages: (i) diffusion of the adsorbate molecules from the bulk solution to the external surface of the adsorbent, (ii) diffusion within the porous structure of the material, and (iii) adsorption on the internal surface of the adsorbent. These all steps determine the overall rate of the adsorption process. While the textural properties of the adsorbent, such as pore size distribution and specific surface area, as well as the quantitative composition of the composite (i.e., the type and amount of active sites), predominantly affect the latter two stages, the first stage is strongly influenced by the particle size and morphology of the adsorbent. A decrease in particle size generally increases the probability of contact between adsorbate molecules and adsorbent particles, thereby facilitating mass transfer from the bulk solution to the external surface of the adsorbent. As it is shown on the SEM images (Figure 6E,J), the SiL2 composite is characterized by a smaller particle size range (10–300 μm vs. 10–400 μm for SiL1) and a narrower their distribution. In the crystal violet adsorption system, this feature is reflected in a higher value of the kinetic parameter log *k_avg_* for SiL2 compared with SiL1, indicating a faster adsorption process. However, no analogous relationship between adsorption rate and particle size distribution was observed for the other two dyes. This suggests that, in these systems, intraparticle diffusion and/or the adsorption step itself contributed more significantly to the overall kinetics than external mass transfer. Since the surface morphologies of both composites are very similar, it is difficult to evaluate the effect of particle morphology on the adsorption behavior.

Table 6 summarizes the kinetic parameters determined using the multiexponential equation, along with the corresponding goodness-of-fit indicators. The multiexponential equation describes the adsorption process within an interval model that accounts for parallel and/or consecutive first-order processes. Comparison of the theoretical curves with the experimental data, together with the goodness-of-fit analysis, demonstrates the high flexibility of the applied model. The quality of the kinetic description is further confirmed by the low values of 1 − R^2^ and SD(c)/c_0_.

Table 7 presents the crystal violet, basic fuchsin, and malachite green adsorption capacities of the SiL2 composite and other adsorbents. Although the adsorption capacity of SiL2 is not outstanding, it is comparable to that of many adsorbents reported previously. While more advanced materials with significantly higher adsorption efficiencies have been developed, their synthesis often requires expensive reagents, equipment, and complex multi-step procedures. In contrast, the silica–lignin composite is composed of naturally derived components and can be prepared using a simple and cost-effective synthesis route. Therefore, despite its relatively low adsorption performance, the material represents a sustainable and environmentally friendly adsorbent consistent with the principles of green chemistry.

### 2.4. Reusability Tests

**Figure 16:**
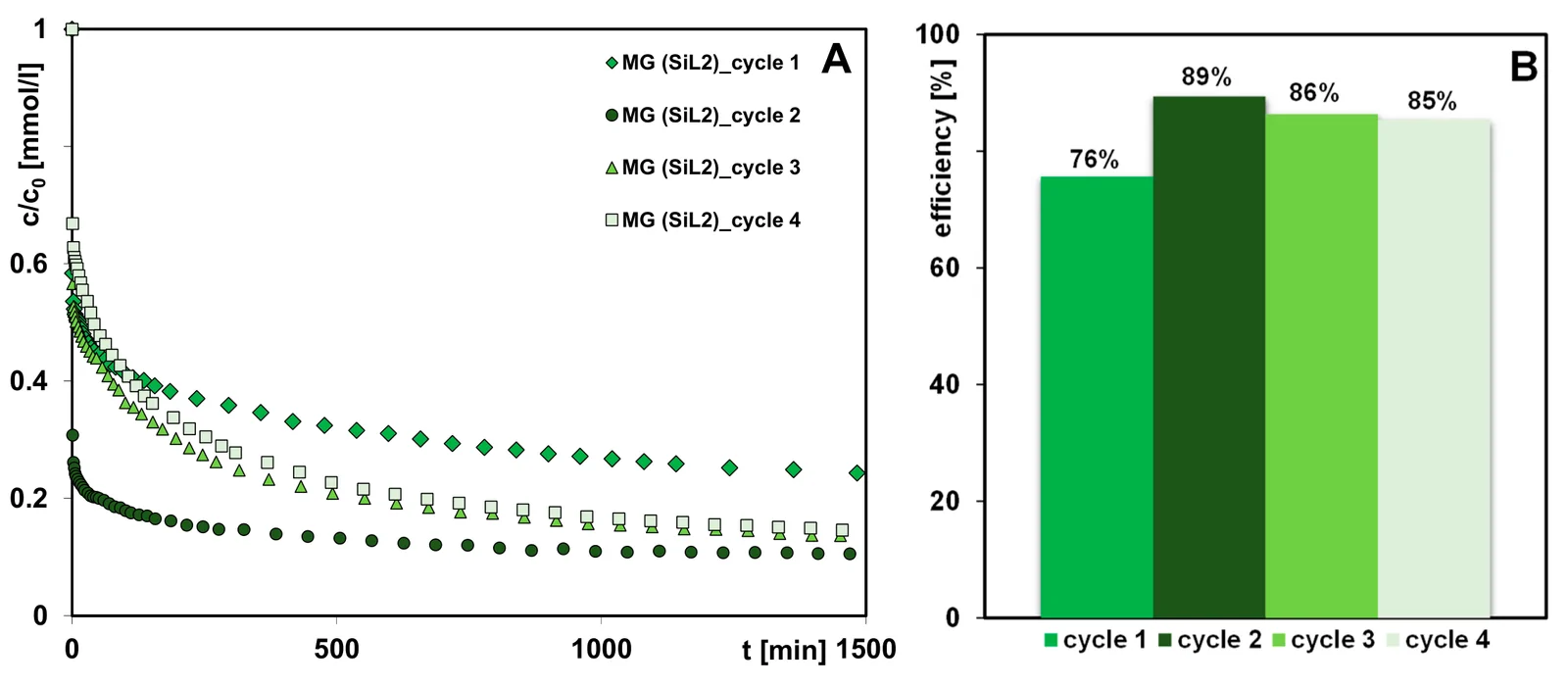
Kinetics of MG adsorption (**A**) and efficiency of reuse of the SiL2 composite over four consecutive process cycles (**B**).

The stability and reusability of the SiL2 composite were evaluated over four consecutive adsorption–desorption cycles using malachite green as the adsorbate and ethanol as the desorption agent (Figure 16A,B). In the first cycle, an adsorption efficiency of 76% was achieved, with approximately 40% of the dye being removed from the solution within the first minute of experiment. Treatment of the spent adsorbent with ethanol markedly improved the purification efficiency, increasing it to 89%, and enhanced the adsorption kinetics. After the first minute, approximately 70% of the dye had been adsorbed, and a near-equilibrium state was reached substantially faster than in the first cycle.

This favorable effect may be attributed to both the properties of the composite formed during synthesis and the applied desorption procedure. Because of the presence of lignin, removal of unreacted organic constituents during composite preparation was performed by washing the material with a water–ethanol mixture rather than by high-temperature calcination. This approach, however, carries the possibility that residual products from the synthesis process may not have been completely removed from the composite. Treatment of the spent material with pure ethanol during dye desorption may have removed residual organic compounds from its porous structure. As a result, the accessible pore volume may have increased and some previously blocked adsorption sites may have become available. In the two subsequent cycles, adsorption efficiency decreased only slightly, reaching 86% and 85%, respectively. At the same time, the initial adsorption kinetics progressively deteriorated: after the first minute, approximately 43% and 32% of the dye, respectively, had been removed from the solution. This effect was most likely associated with incomplete desorption of the dye during the preceding adsorption–desorption cycles. Nevertheless, the adsorption efficiency achieved in cycles 2–4 remained considerably higher than that observed for the initial composite. These results demonstrate the high stability of the SiL2 composite in contact with the applied desorption agent and confirm its potential for repeated use in the removal of cationic dyes from aqueous solutions.

## 3. Materials and Methods

### 3.1. Chemicals and Materials

#### 3.1.1. Chemicals

Alkali lignin, kraft, Pluronic P123 and tetraethyl orthosilicate (TEOS, ≥99.0%) were purchased from Sigma-Aldrich (Poznań, Poland). Hydrochloric acid (35–38 wt%) and ethanol (96%) were supplied by POCh (Poznań, Poland). Crystal violet (90%) was purchased from Thermo Scientific Chemicals (Waltham, Massachusetts, USA), basic fuchsin (90%) from Warchem (Zakret, Poland), and malachite green (99%) from Hadron Scientific (Kielce, Poland).

#### 3.1.2. Synthesis of Silica–Lignin Composites

Two silica–lignin composites, differing in the amount of added lignin, were synthesized by a modified sol–gel method. The materials were synthesized in acidic solution by adding tetraethyl orthosilicate (TEOS) as a silica precursor. The synthesis scheme was as follows: 4 g of Pluronic P123 was dissolved in 150 mL of 1.6 M HCl and heated at 308 K for 1 h with stirring (700 rpm). Then, 0.5 or 1.5 g of lignin was added and heating and stirring were continued for 0.5 h. After that, 9.2 mL of TEOS was added and the mixture was kept under the same conditions for 24 h. After synthesis, the obtained samples were aged at 343 K for 48 h, filtered, washed with distilled water, and dried at 343 K for 24 h. Then, 200 mL of ethyl alcohol was added to both samples and they were subjected to alcohol extraction in a shaker. After 48 h, the samples were filtered under vacuum and subjected to another alcohol extraction under the same conditions as before. After another 48 h, the samples were filtered again and dried at 70 °C for 24 h.

The materials received were marked as follows: SiL1 (0.5 g of lignin) and SiL2 (1.5 g of lignin).

### 3.2. Textural, Structural, Surface and Thermal Characteristics of Silica–Lignin Composites

The parameters characterizing the textural properties of silica–lignin composites and lignin were determined based on nitrogen adsorption–desorption isotherms measured at 77 K using an ASAP 2020 analyzer (Micromeritics, Norcross, GA, USA). Before analysis, samples were degassed at 60 °C under vacuum (1 mmHg) for 24 h. The specific surface area, *S_BET_*, was calculated by the Brunauer–Emmett–Teller (BET) method [65]. The total pore volume, *V_t_*, was estimated from the amount of adsorbed nitrogen at relative pressure *p*/*p_s_* = 0.98. The micropore volume, *V_mic_*, and surface area, *S_mic_*, were determined by the t-plot method. The external surface area, *S_ext_*, and mesopore volume, *V_mes_*, were determined using the α_s_ method with LiChrospher Si-1000 silica as a standard [66]. The pore size distributions were calculated from the adsorption branches of isotherms using the Barrett–Joyner–Halenda (BJH) model with cylindrical pore geometry and the KJS correction [67]. Moreover, to determine the micropore distribution functions, the Horvath-Kawazoe (HK) and DFT methods were applied.

The surface morphology of the samples was characterized by scanning electron microscopy (SEM) using a Quanta™ 3D FEG microscope (FEI Company, Hillsboro, OR, USA) at an accelerating voltage of 30 kV.

X-ray diffraction measurements were carried out using an EMPYREAN diffractometer (PANalytical, Almelo, The Netherlands) operating in Bragg–Brentano reflection geometry. The instrument was equipped with a sealed X-ray tube with a Cu anode (Cu Kα radiation, *λ* = 1.5406 Å) and a divergence slit ½ on the incident beam side. The generator was set to an acceleration voltage of 40 kV and a tube current of 40 mA. Powder samples of the silica–lignin composites were gently ground in an agate mortar to obtain a homogeneous particle size distribution and then spread onto a flat, low-background sample holder to form a smooth, compact layer, minimizing preferred orientation and surface roughness effects. The sample surface was aligned in the focal plane of the diffractometer to ensure optimal reflection conditions. Diffraction patterns were recorded in the 2θ range from 10° to 80° in continuous scan mode. A typical step size of 0.02° 2θ and a counting time of 0.5–1 s per step (or an equivalent scan speed) were used to obtain a satisfactory signal-to-noise ratio while maintaining reasonable measurement time. A Ni filter or appropriate monochromator was applied on the diffracted beam to reduce Kβ radiation, and a solid-state or PIXcel detector (depending on instrument configuration) was used for data acquisition. X-ray Photoelectron Spectroscopy (XPS) allowed determination of surface chemistry and electronic states of the atoms. In the measurements the Ultra High Vacuum (UHV) multi-chamber analytical system (Prevac, Rogów, Poland) equipped with Scienta R4000 hemispherical analyzer was used. The CasaXPS software (v 2.3.17) was applied for data analysis.

Chemical mapping was performed with a Nicolet iN10 MX FTIR microscope equipped with a motorized XY stage and a liquid-nitrogen-cooled mercury cadmium telluride (MCT) detector in reflection mode. Chemical distribution maps were generated and processed using OMNIC Specta™ software (version 8.1) based on spectral correlation with reference spectra of the silica carrier and the pure dye. Transmission FTIR spectra were obtained using a FTIR-Nicolet 8700A spectrometer (Thermo Scientific, Waltham, MA, USA).

The acid-base properties of silica–lignin composites were determined by potentiometric titration. Measurements were performed in pre-acidified suspensions of the composites at a temperature of 25 °C. The suspensions were titrated with an NaOH solution using an automatic burette (Dosimat 765, Metrohm, Herisau, Switzerland), and pH changes were recorded using a pH meter (PHM240, Radiometer, Copenhagen, Denmark). Based on the recorded pH changes as a function of the volume of added titrant, the surface charge density and the point of zero charge for the materials under study were determined.

Thermal analysis was carried out on a STA 449 Jupiter F1 instrument (NETZSCH, Selb, Bavaria, Germany). Thermal analysis was carried out on a STA 449 Jupiter F1 instrument (NETZSCH, Selb, Bavaria, Germany). Samples were heated from 30 °C to 1000 °C at a heating rate of 10 °C·min^−1^, with a gas flow rate of 50 mL·min^−1^. Measurements were conducted in an atmosphere of either synthetic air or nitrogen (6.0 purity, Air Liquide) using Al_2_O_3_ crucibles and a TG-DSC setup equipped with an S-type thermocouple. An empty Al_2_O_3_ crucible served as a reference. The average sample masses were 8.18 ± 0.13 mg for the air atmosphere and 14.10 ± 0.11 mg for the nitrogen atmosphere. Before measuring the samples in the atmosphere of a given gas, correction measurements were performed for empty crucibles, and the temperature and sensitivity calibration was checked using In and Al samples. Gaseous decomposition products were analysed via infrared and mass spectra, recorded using a Tensor 27 FTIR spectrometer (Bruker, Ettlingen, Germany) equipped with a TGA–IR interface, and a QMS 403 D Aëolos quadrupole mass spectrometer (NETZSCH, Selb, Germany). Data acquisition and processing were carried out using NETZSCH Proteus^®^ software (version 6.1), OPUS (version 7.2), and NIST MS Search 2.0.

### 3.3. Dye Adsorption Measurements

The adsorption of crystal violet (CV), basic fuchsin (BF), and malachite green (MG) from aqueous media onto silica–lignin composite materials was investigated using a batch adsorption technique. For each experiment, approximately 0.05 g of adsorbent was placed in an Erlenmeyer flask, followed by the addition of 25 mL of dye solution at a predetermined initial concentration (0.017–0.177 mmol/L). The ionic strength and pH were not adjusted during the experiments. The initial pH values of the stock dye solutions were 6.7 for CV, 6.4 for BF, and 6.8 for MG. The adsorption process was performed at 25 °C under continuous agitation at 120 rpm for a period of 5 days to get equilibrium. Upon completion of the adsorption stage, the suspensions were filtered, and the residual dye concentrations were determined by UV-Vis spectrophotometry using a Cary 4000 spectrophotometer (Varian Inc., Melbourne, VIC, Australia). The analysis of the equilibrium data was based on the Generalized Langmuir (GL) equation [68,69,70,71]:

(1) $$\theta ={\left[\frac{{\left(Kc_{eq}\right)}^{n}}{1+{\left(Kc_{eq}\right)}^{n}}\right]}^{\frac{m}{n}}$$

where *θ* = *a_eq_*/*a_m_* is the global adsorption isotherm; *a_m_* is the adsorption capacity; *m*, *n* are the heterogeneity parameters; and *K* is the equilibrium constant. The GL isotherm was derived based on a general integral equation describing localized adsorption on energetically heterogeneous solids. The energetic heterogeneity is a feature of the entire adsorption system consisting of adsorbent and adsorbate. Energy distribution functions define the shape of the energy distribution, while its position is determined by a characteristic or mean energy. The width of the energy distribution provides a general indication of the extent to which adsorption sites differ from one another in terms of their adsorption energy. The heterogeneity parameters *m* and *n* characterize the shape of the distribution function of adsorption energy differences. The equilibrium constant, *K*, determines the position of the distribution function on the energy axis.

Kinetic studies were carried out using the continuous registration method of the adsorbate solution spectra [72,73]. The experiments were conducted in a glass vessel maintained at 25 °C, into which 0.1 g of adsorbent and 50 mL of a dye solution with a concentration of 0.035 mmol/L were introduced. At specified time intervals, samples were automatically withdrawn from the system and directed to a flow-through cuvette, where UV-Vis spectra were recorded using a Cary 100 spectrophotometer (Varian Inc., Melbourne, VIC, Australia). Throughout the entire experiment, the suspension was subjected to continuous mechanical stirring at a speed of 120 rpm. Changes in absorbance at the maximum of the characteristic absorption band of the dye were converted into changes in its concentration as a function of time. The obtained data were further analyzed using: (i) the multi-exponential equation (m-exp) [74]:

(2) $$c=\left(c_{o}-c_{eq}\right)\sum_{i=1}^{n}f_{i}exp\left(-k_{i}t\right)+c_{eq}$$

(3) $$c=c_{0}-c_{o}u_{eq}\sum_{i=1}^{n}f_{i}\left[1-exp\left(-k_{i}t\right)\right]$$

where *i* is the term of the m-exp equation, *k_i_* is the rate coefficient, and *u_eq_* = 1 − *c_eq_*/*c_o_* is the relative loss of adsorbate from the solution.

## 4. Conclusions

High-resolution X-ray photoelectron spectroscopy (XPS) analysis indicates the presence of lignin-derived organic functionalities within the surface region of the silica particles, confirming successful surface modification while preserving the structural integrity of the silica matrix. The observed shift in the O 1s core-level toward lower binding energy suggests changes in the local electronic environment of oxygen atoms, which may result from interactions between oxygen-containing functional groups of lignin and the silica surface.

The high adsorption efficiency of CV can be attributed to synergistic effects between the dye’s intrinsic structural features (e.g., regular molecular geometry and capacity for multipoint interactions) and the heterogeneous yet functionally complementary surface chemistry of the silica–lignin composite. Regions exhibiting the greatest lignin and silica contributions coincide with the highest CV signal intensities, indicating that the co-localization of both composite constituents provides the most favorable microenvironment for the formation of an ordered adsorbed dye layer.

In contrast, the comparatively lower uptake of BF relative to CV, as supported by the isotherm data, is likewise captured in the correlation maps. The limited BF signal in silica-rich domains suggests that this dye does not effectively exploit the porous architecture of the composite; instead, it preferentially accumulates on lignin-rich surface sites. This spatial distribution is consistent with surface-level agglomeration of BF molecules rather than their ordered organization within the pore network.

## Figures and Tables

**Table 1:** The textural parameters of silica–lignin composites and lignin.

Composite	*S_BET_* ^1^ [m^2^/g]	*V_t_* ^2^ [cm^3^/g]	*α_s_* Plot *S_ext_* ^3^ [m^2^/g]	*α_s_* Plot *V_p_* ^4^ [cm^3^/g]	*t*-Plot *S_mic_* ^5^ [m^2^/g]	*t*-Plot *V_mic_* ^6^ [cm^3^/g]	*V_p_*/*V_t_* ^7^	*D_BJH ads_* ^8^ [nm]	*D_mo_* H-K ^9^ [nm]
**SiL1**	616	0.76	48	0.58	123	0.05	0.76	5.13	0.81
**SiL2**	444	0.60	24	0.55	55	0.02	0.92	5.26	0.82
**L**	2.94	0.01	-	-	-	-	-	10.07	-

^1^ BET specific surface area, determined from experimental data within a relative pressure range (*p*/*p_s_*) of approximately 0.058–0.20, where *p* denotes the equilibrium pressure and *p_s_* represents the saturation pressure of nitrogen ^2^ Total pore volume calculated from the amount of nitrogen adsorbed at *p*/*p_s_* = 0.98. ^3^ External surface area determined on the basis of *α_s_*-plot (LiChrospher Si-1000 silica was used as a standard). ^4^ Primary mesopore volume determined on the basis of *α_s_*-plot. ^5^ Micropore volume determined by the t-plot method. ^6^ Micropore surface area determined by the t-plot method. ^7^ Mesopore contribution. ^8^ Pore diameter estimated based on the maximum of the pore size distribution (PSD) according to the BJH theory, determined from adsorption data. ^9^ Micropore diameter estimated based on the HK theory.

**Table 3:** Thermal behavior of studied samples (T_max_—the maximum degradation).

Adsorbent (Atmosphere)	Temperature Range [°C]	Mass Loss [%]	Total Mass Change [%]	DTG *T_max_* [°C]	DSC *T_max_* [°C] (endo↑; exo↓)
SiL1 (N_2_)	35–190	2.6	19.1	82	82↑
	190–636	13.9		306, 446,	421↑, 498↑, 596↓
	636–1000	2.6		–	–
SiL2 (N_2_)	35–142	2.0	25.5	75	75↑
	142–607	16.5		409	519↓
	607–1000	7.0		–	–
L (N_2_)	35–154	3.4		91, 136	96↑
	154–573	30.6	58.8	351	186 (Tg), 355↓, 447↓, 467↓
	573–800	13.7		738,	744↑,
	800–906	6.1		843	864↑
	906–1000	5			
SiL1 (air)	35–126	1.4		72	76↑
	126–300	7.5	19.5	253	253↓
	300–566	8.7		343, 493	357↓, 485↓
	566–1000	1.9		–	588↑
SiL2 (air)	35–126	1.3	28.8	68	75↑
	126–335	9.8		285	283↓
	335–554	15.5		393, 472	360↓, 476↓
	554–1000	2.2		–	–↑
L (air)	35–154	3.5	75.5	82	89↑
	154–625	33.2		295, 359	297↓, 362↓
	625–1000	38.8		778	780↓

**Table 4:** Physicochemical properties of the triarylmethane dyes.

Dye Code/Structure	Molecular Weight [g/mol]	Ionization Constant, p*K_a_*	Water Solubility [%]	Maximal Projection Area, *A_max_* [Å^2^]	Van der Waals Volume, *V* [Å^3^]
CV 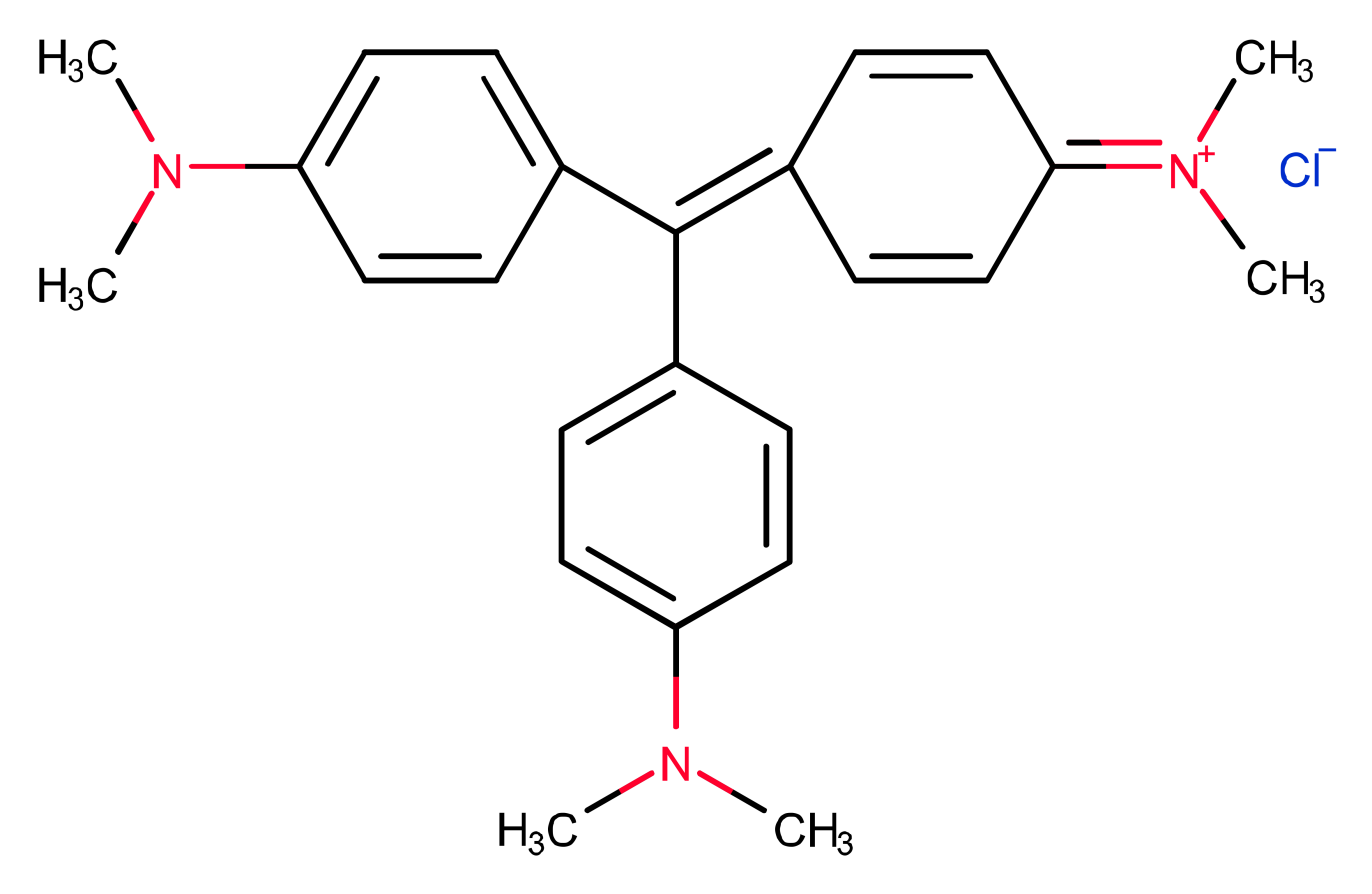	407.99	1.2; 5.4	1.7	116	372
BF 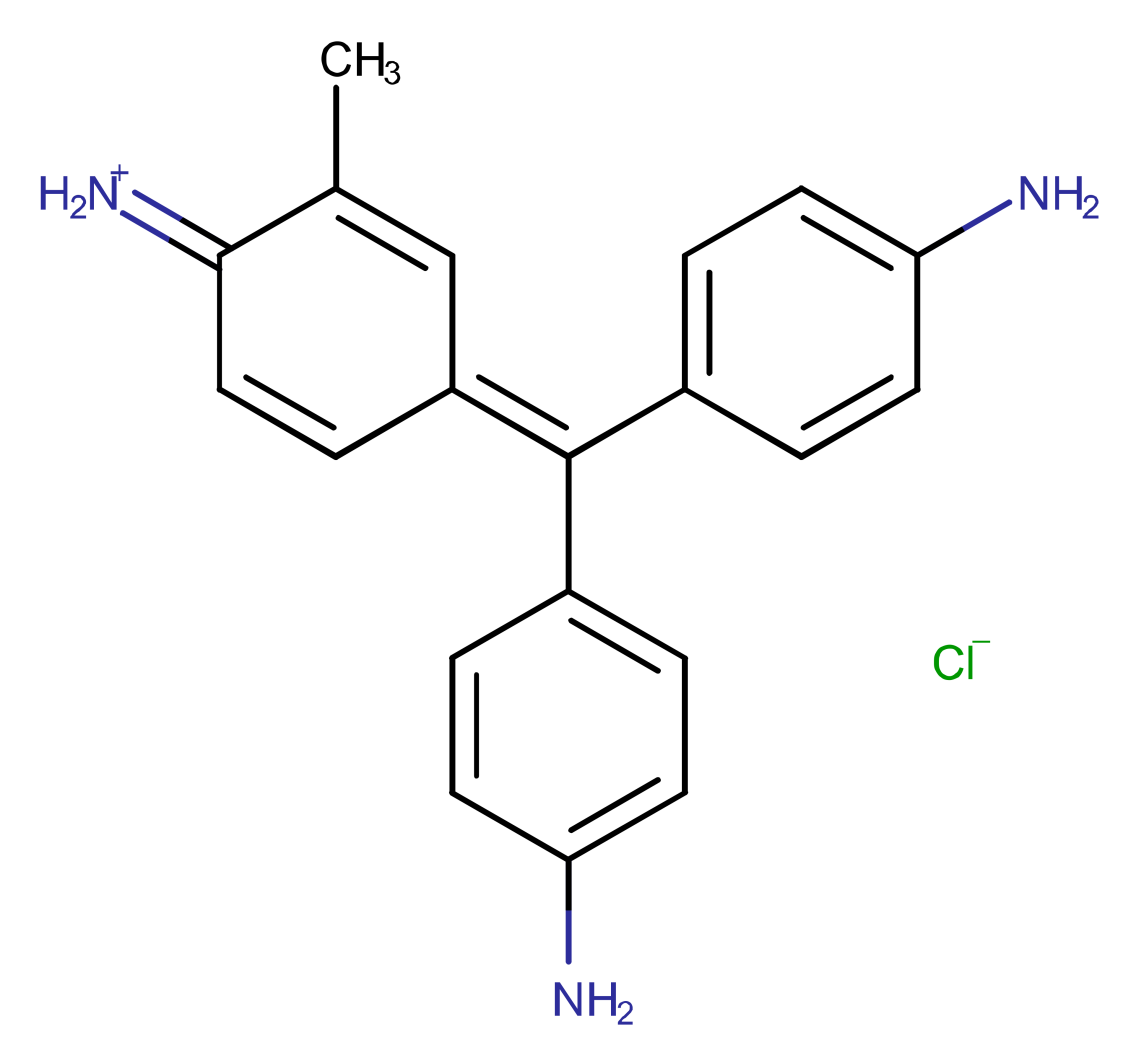	337.86	2.1	0.5	86	281
MG 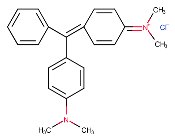	364.91	6.9	4	99	326

**Table 5:** The parameters of the Generalized Langmuir equation for dye (silica–lignin composite) systems.

Adsorption System	*a_m_* [mmol/g]/[mg/g]	*m*/*n*	log *K*	R^2^	SD(a)
CV (SiL1)	0.033/13.5	1/0.91	1.68	0.991	7.91 × 10^−4^
CV (SiL2)	0.041/16.7	1/0.62	2.27	0.977	1.49 × 10^−3^
BF (SiL1)	0.025/8.4	1/0.90	1.63	0.954	1.31 × 10^−3^
BF (SiL2)	0.032/10.8	1/0.98	1.72	0.983	1.13 × 10^−3^
MG (SiL1)	0.013/4.7	1/1	1.80	0.993	2.17 × 10^−4^
MG (SiL2)	0.018/6.6	1/1	1.90	0.941	1.06 × 10^−3^

**Table 6:** Comparison of the kinetic parameters determined from the multi-exponential equation.

Kinetic System	*i*	*f_i_*	log *k_i_*	log *k_avg_*	*t*_0.5,*i*_ [min]	*t*_0.5 *avg*_ [min]	*t*_50%_ [min]	*u_eq_*	SD (c/c_o_)	1 − R^2^
CV (SiL1)	1	0.90	0.44	0.37	0.25	0.29	0.38	0.85	0.20%	2.0 × 10^−4^
	2	0.03	−1.32		14					
	3	0.07	−2.85		488					
CV (SiL2)	1	0.94	0.54	0.50	0.20	0.22	0.26	0.90	0.09%	4.0 × 10^−5^
	2	0.02	−1.00		7					
	3	0.04	−2.53		237					
BF (SiL1)	1	0.88	0.63	0.55	0.16	0.20	0.36	0.72	0.18%	2.8 × 10^−4^
	2	0.07	−1.34		15					
	3	0.04	−2.89		540					
BF (SiL2)	1	0.83	0.59	0.47	0.18	0.24	0.33	0.82	0.17%	1.6 × 10^−4^
	2	0.08	−0.80		4					
	3	0.09	−2.74		379					
MG (SiL1)	1	0.57	0.47	0.01	0.23	0.69	125	0.67	0.58%	2.0 × 10^−3^
	2	0.14	−1.65		31					
	3	0.29	−2.81		442					
MG (SiL2)	1	0.60	0.34	−0.07	0.32	0.82	13	0.78	0.25%	3.0 × 10^−4^
	2	0.12	−1.50		22					
	3	0.28	−2.80		441					

**Table 7:** Comparison of the adsorption performance of various adsorbents towards the dye pollutans.

Adsorbate	Adsorbent	Adsorption Capacity, a_m_ [mg/g]	Reference
crystal violet	silica–lignin composite	16.7	this paper
	carbon embedded silica from rice husk (CES)	18.8	[51]
	activated carbon from lemon wood	23.6	[52]
	nascent rice husk	24.5	[53]
	lignin copper ferrite	34.1	[54]
	lignin/PVA hydrogel	190	[55]
basic fuchsin	silica–lignin composite	10.8	this paper
	alkali-activated diatomite	9.8	[56]
	*Eucalyptus bark* based magnetic composite	9.6	[57]
	deoiled soya	13.5	[58]
	MgO/BaCO_3_/BaCrO_4_/C nanocomposite	376	[59]
malachite green	silica–lignin composite	6.6	this paper
	humic acid	6.0	[60]
	*Arundo donax* root carbon	8.7	[61]
	bentonite	7.7	[62]
	activated rice husk	12.0	[63]
	aminated lignin/cellulose/Fe_3_O_4_	155	[64]

## Data Availability

The raw data supporting the conclusions of this article will be made available by the authors on request.

## Supplementary Materials

The following supporting information can be downloaded at: https://www.mdpi.com/article/10.3390/ijms27219954/s1.
