# Supplementary material for: Silica–Lignin Composites—Insight into Physicochemical Properties and Adsorption Mechanisms
Source: Int J Mol Sci. 2026 Nov 6;27(21):9954. doi: 10.3390/ijms27219954 (PMC13763654; doi:10.3390/ijms27219954)
Supplement: Supplementary file 1 [file ijms-27-09954-s001.zip › ijms-4655375-supplementary.pdf]

Supplementary Materials

Silica–Lignin Composites – Insight into Physicochemical Properties and Adsorption Mechanisms

Magdalena Blachnio*, Malgorzata Zienkiewicz-Strzalka*, Zofia Juchniewicz, Dariusz Sternik and Anna Derylo-Marczewska*

Department of Physical Chemistry, Institute of Chemical Sciences, Maria Curie-Sklodowska University, Maria Curie-Sklodowska Square 3, 20-031 Lublin, Poland; malgorzata.zienkiewicz-strzalka@mail.umcs.pl (M.Z.-S.); dariusz.sternik@umcs.pl (D.S.); zofia_juchniewicz@interia.pl (Z.J.)

* Correspondence: magdalena.blachnio@poczta.umcs.lublin.pl (M.B.); anna.derylo-marczewska@mail.umcs.pl (A.D.-M.)

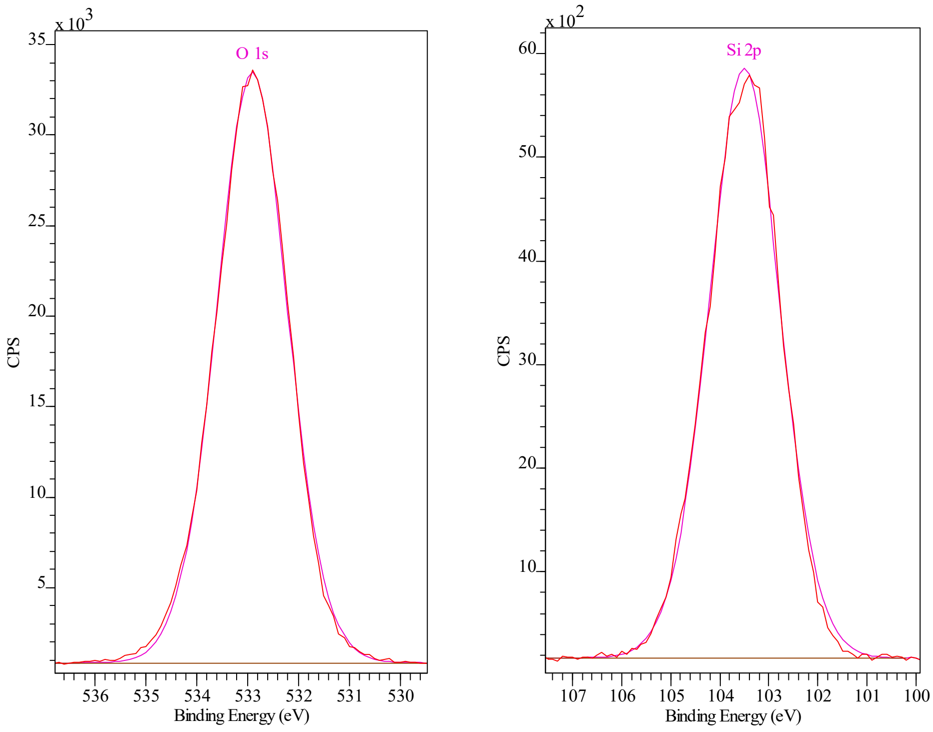

Figure S1. High-resolution XPS spectra ofO1s and Si2p atoms for SiL2 sample.

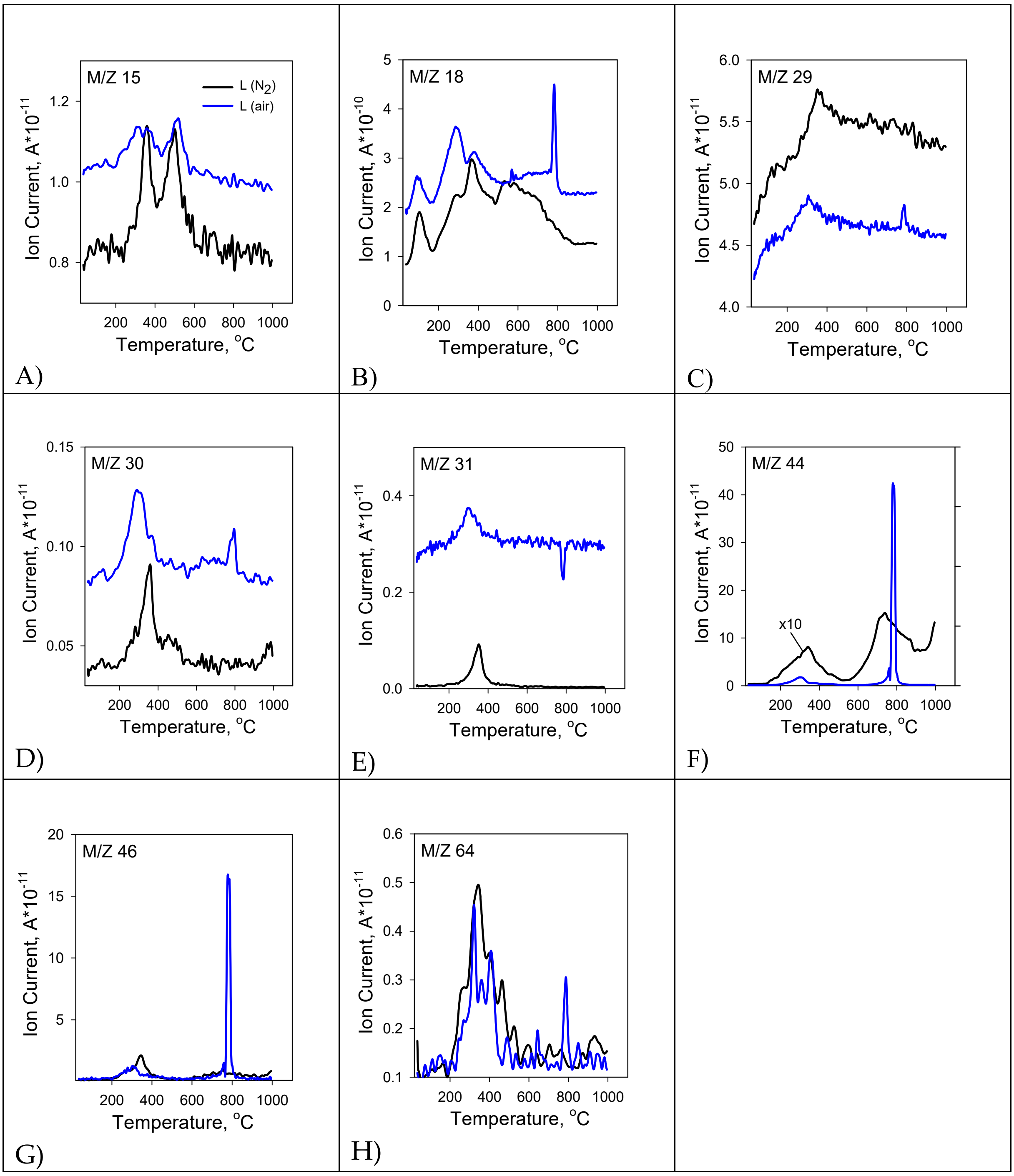

Figure S2. Temperature-dependent QMID ion current profiles at selected m/z values, collected during the heating of lignin in nitrogen (black line) and in synthetic air (blue line).

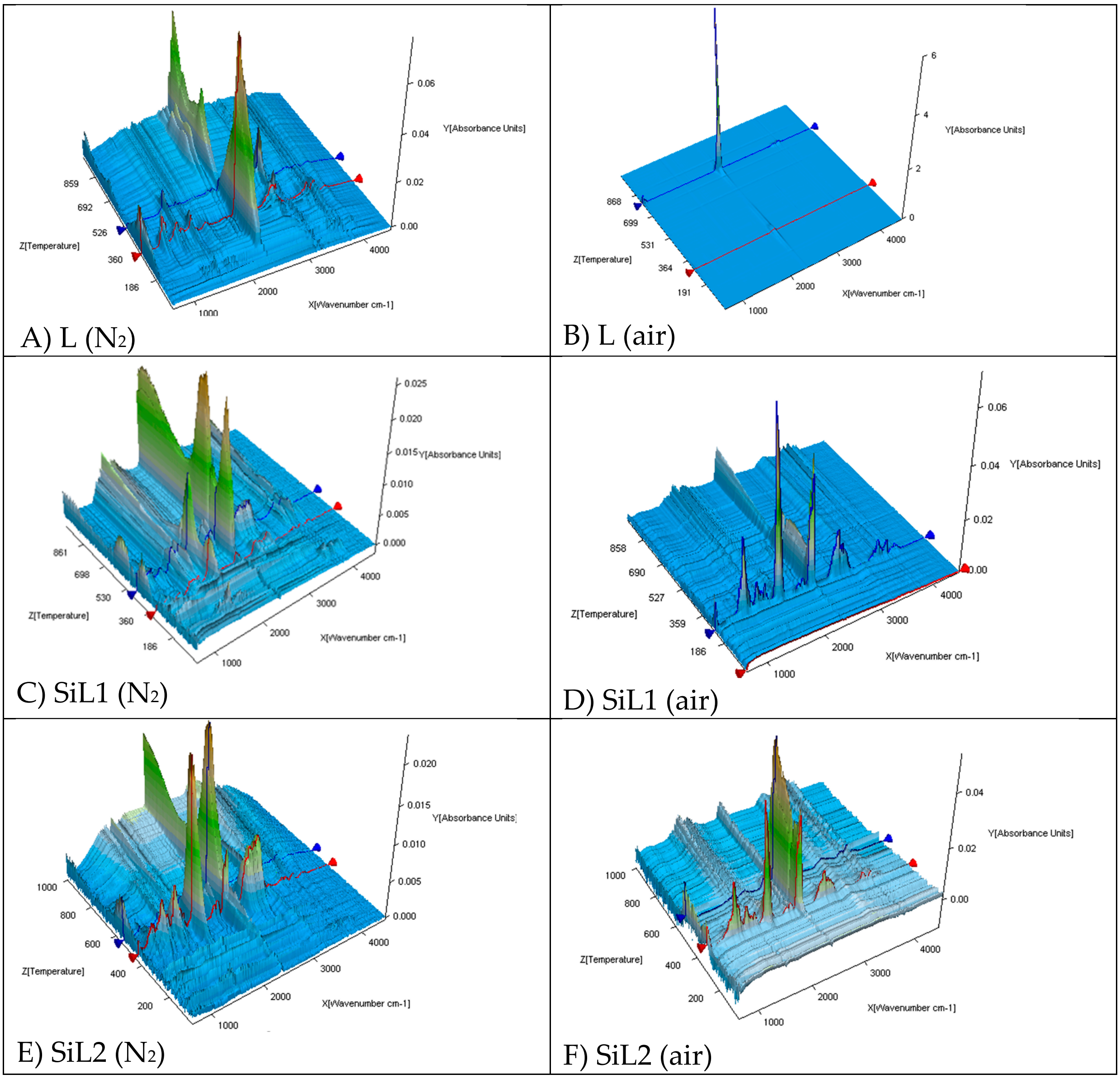

Figure S3. 3D diagrams of FTIR spectra of volatiles formed during the heating of lignin (A,B) and of composites SiL1 (C,D) and SiL2 (E,F) in nitrogen (A,C,E) and air (B,D,F).

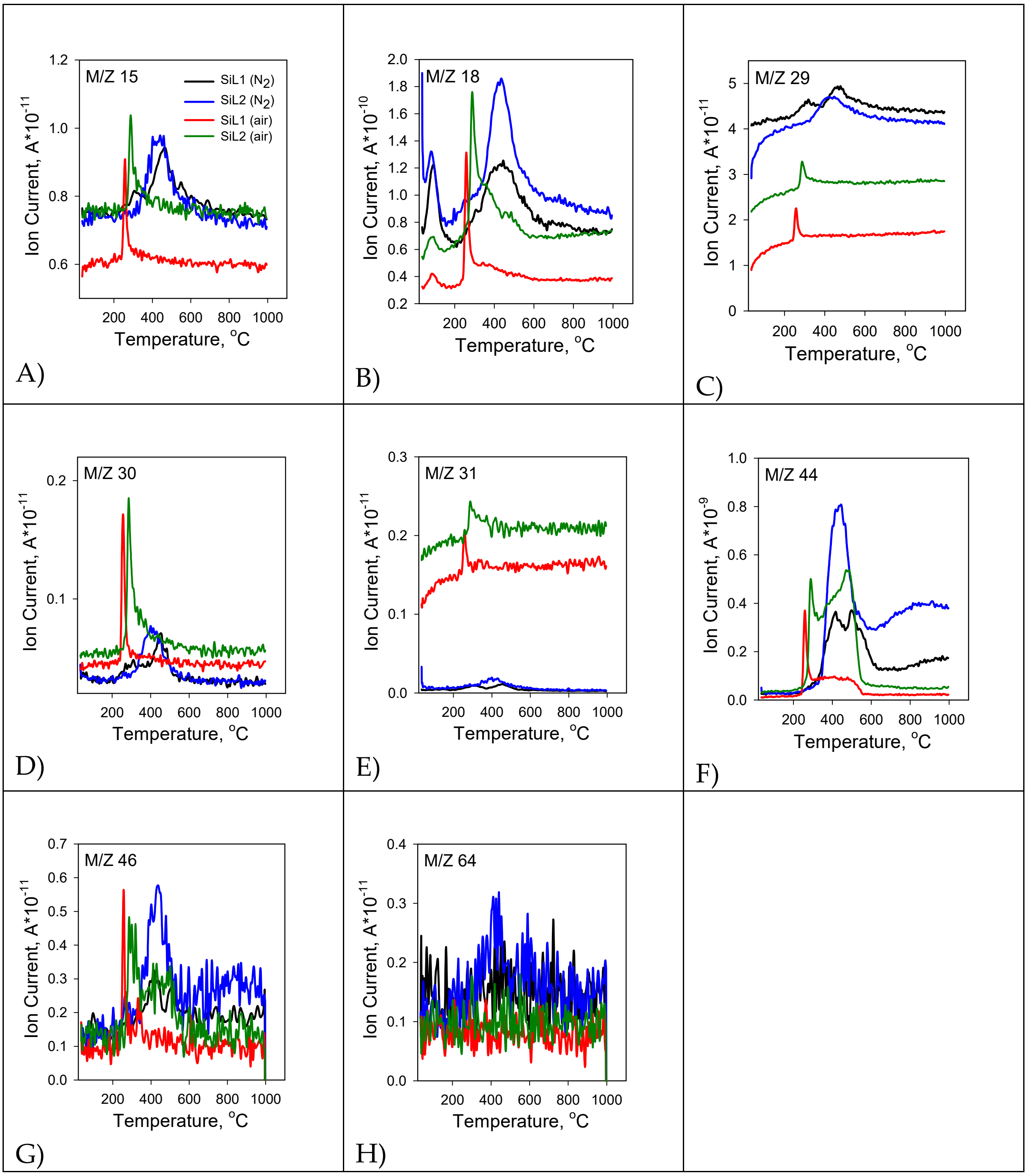

Figure S4. Temperature–dependent QMID ion current profiles at selected m/z values, collected during the heating of composites in nitrogen and in synthetic air.

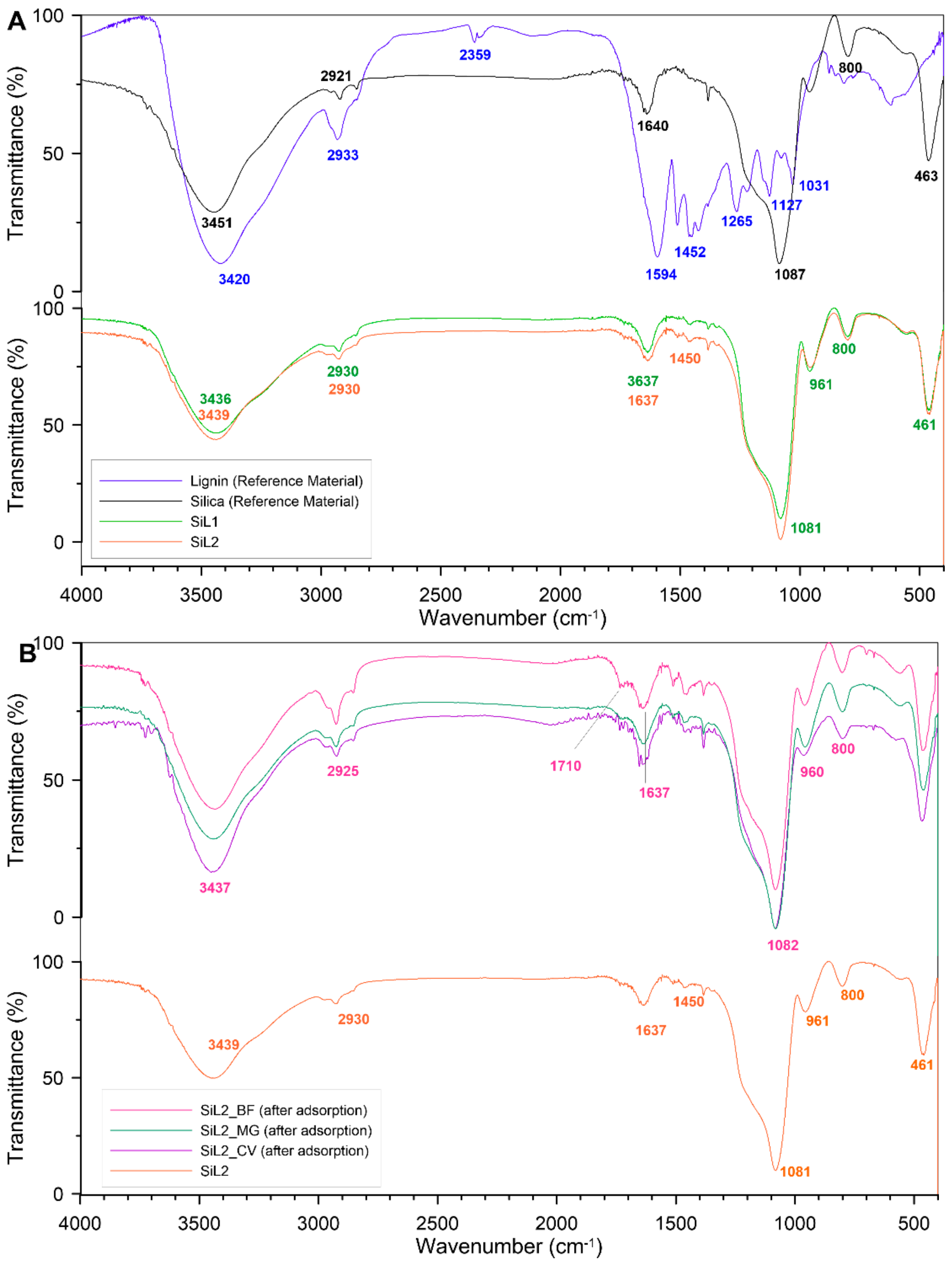

Figure S5. (A) Fourier-transform infrared (FTIR) spectra of lignin and silica reference materials, compared with those of two lignin–silica composites (SiL1 and SiL2). (B) FTIR spectra of lignin–silica materials following dye adsorption, compared with the corresponding SiL2 spectra acquired prior to the adsorption experiment.

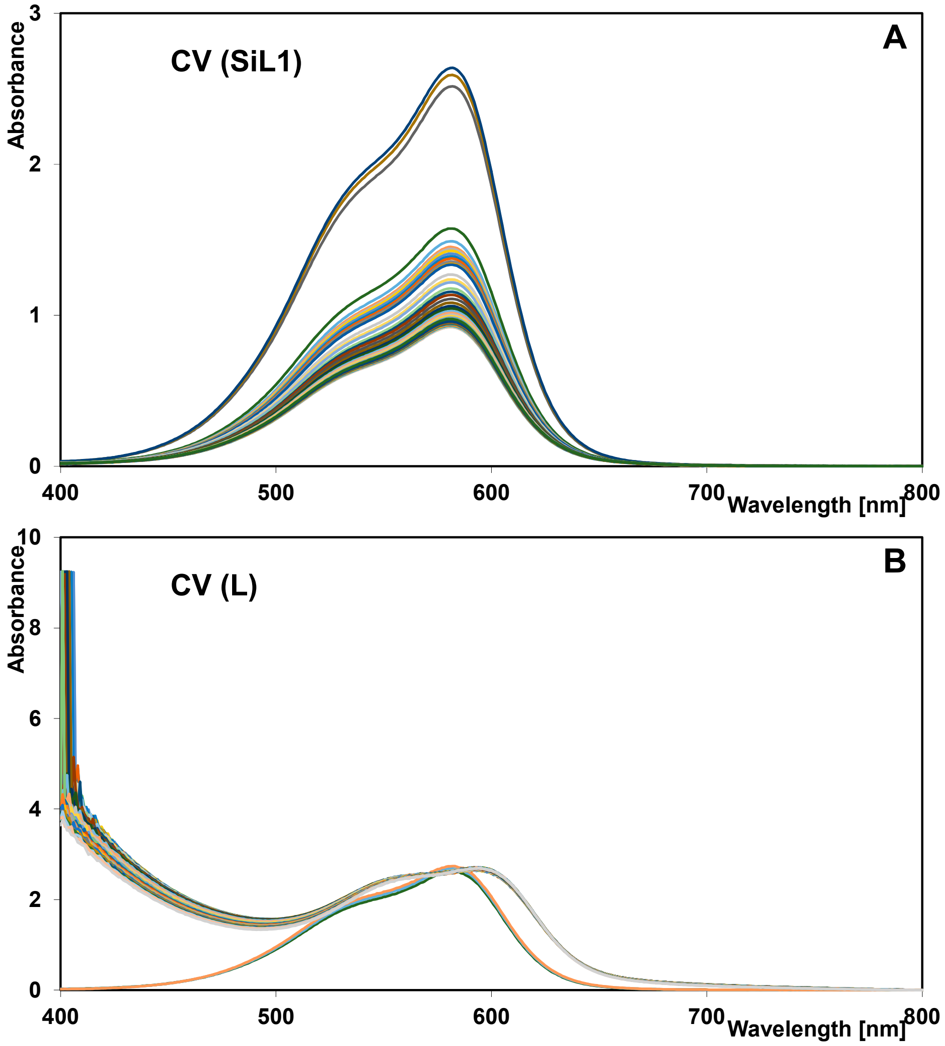

Figure S6. A series of Vis absorption spectra obtained for the CV (SiL1) (A) and CV (L) (B) system.
